# A novel *FLI1* exonic circular RNA promotes metastasis in breast cancer by coordinately regulating TET1 and DNMT1

**DOI:** 10.1186/s13059-018-1594-y

**Published:** 2018-12-11

**Authors:** Naifei Chen, Gang Zhao, Xu Yan, Zheng Lv, Hongmei Yin, Shilin Zhang, Wei Song, Xueli Li, Lingyu Li, Zhonghua Du, Lin Jia, Lei Zhou, Wei Li, Andrew R. Hoffman, Ji-Fan Hu, Jiuwei Cui

**Affiliations:** 1 Stem Cell and Cancer Center, The First Hospital of Jilin University, 71 Xinmin Street, Changchun, 133021 Jilin China; 2 Department of Breast Cancer Surgery, The First Hospital of Jilin University, 71 Xinmin Street, Changchun, 133021 Jilin China; 3 Department of General Internal Medicine, The First Hospital of Jilin University, 71 Xinmin Street, Changchun, 133021 Jilin China; 4 Stanford University Medical School, Palo Alto Veterans Institute for Research, Palo Alto, CA 94304 USA

**Keywords:** Circular RNA, *FLI1*, Breast cancer, DNA methylation, TET1, DNMT1, Tumor

## Abstract

**Background:**

Friend leukemia virus integration 1 (FLI1), an ETS transcription factor family member, acts as an oncogenic driver in hematological malignancies and promotes tumor growth in solid tumors. However, little is known about the mechanisms underlying the activation of this proto-oncogene in tumors.

**Results:**

Immunohistochemical staining showed that FLI1 is aberrantly overexpressed in advanced stage and metastatic breast cancers. Using a CRISPR Cas9-guided immunoprecipitation assay, we identify a circular RNA in the FLI1 promoter chromatin complex, consisting of FLI1 exons 4-2-3, referred to as FECR1.Overexpression of FECR1 enhances invasiveness of MDA-MB231 breast cancer cells. Notably, FECR1 utilizes a positive feedback mechanism to activate FLI1 by inducing DNA hypomethylation in CpG islands of the promoter. FECR1 binds to the FLI1 promoter in cis and recruits TET1, a demethylase that is actively involved in DNA demethylation. FECR1 also binds to and downregulates in trans DNMT1, a methyltransferase that is essential for the maintenance of DNA methylation.

**Conclusions:**

These data suggest that FECR1 circular RNA acts as an upstream regulator to control breast cancer tumor growth by coordinating the regulation of DNA methylating and demethylating enzymes. Thus, FLI1 drives tumor metastasis not only through the canonical oncoprotein pathway, but also by using epigenetic mechanisms mediated by its exonic circular RNA.

**Electronic supplementary material:**

The online version of this article (10.1186/s13059-018-1594-y) contains supplementary material, which is available to authorized users.

## Introduction

The ETS gene family of transcription factors share a common ETS DNA binding domain responsible for sequence-specific DNA recognition on target promoters, and they play critical roles in controlling cell proliferation, transformation, apoptosis, and tumorigenesis [1–3]. Among the ETS family members, Friend leukemia virus integration 1 (*FLI1*) was first identified as a proto-oncogene activated by proviral integration in F-MuLV-induced erythroleukemias [4]. *FLI1* is preferentially expressed in hematopoietic cells and tissues, where it regulates hematopoietic stem cell self-renewal and differentiation [5, 6]. Aberrant expression of *FLI1* may act as a critical driver in the development of hematological malignancies [7–10]. Overexpression of *FLI1* in erythroblasts causes inhibition of differentiation and ultimately the development of pre-T cell lymphoblastic leukemia/lymphoma [11]. In addition to erythroleukemia, the aberrantly deregulated *FLI1* is also associated with other hematological malignancies, including pre-T cell lymphoblastic lymphoma, acute myeloid leukemia, and B cell lymphomas [1].

Recent studies have shown that *FLI1* is also aberrantly expressed in some solid tumors, including Ewing sarcoma [12, 13], metastatic melanomas [14], nasopharyngeal carcinoma [13], and non-Ewing soft tissue tumors [15]. A series of clinical studies from our lab showed that *FLI1* is overexpressed in breast cancers [16] and lung cancers [17]. In patients with breast cancer, expression of *FLI1* is strongly correlated with advanced stage, poor differentiation, and lymph node metastasis. In highly metastatic human breast cancer cells, knockdown of *FLI1* significantly attenuated tumor metastasis through the Rho GTPase pathway [16]. In non-small cell lung cancer, the *FLI1* expression score is associated with the stage of SCLC. In these cells, *FLI1* promoted tumor metastasis by activating the miR-17-92 cluster family [17].

However, little is known about the molecular mechanisms underlying the aberrant activation of *FLI1* in these solid tumors. In this study, we harnessed a novel CRISPR Cas9-guided promoter immunoprecipitation (CasIP) assay to identify the molecular components that interact with the *FLI1* promoter. We hypothesized that these components might actively participate in the control of *FLI1* in the development of breast cancers. Using this CasIP assay, we identified FECR1, a *FLI1* exonic circular RNA that binds to the *FLI1* promoter and epigenetically activates *FLI1* in breast cancer cells.

## Results

### CasIP identifies a novel FLI1 exonic circular RNA

**Fig. 1:**
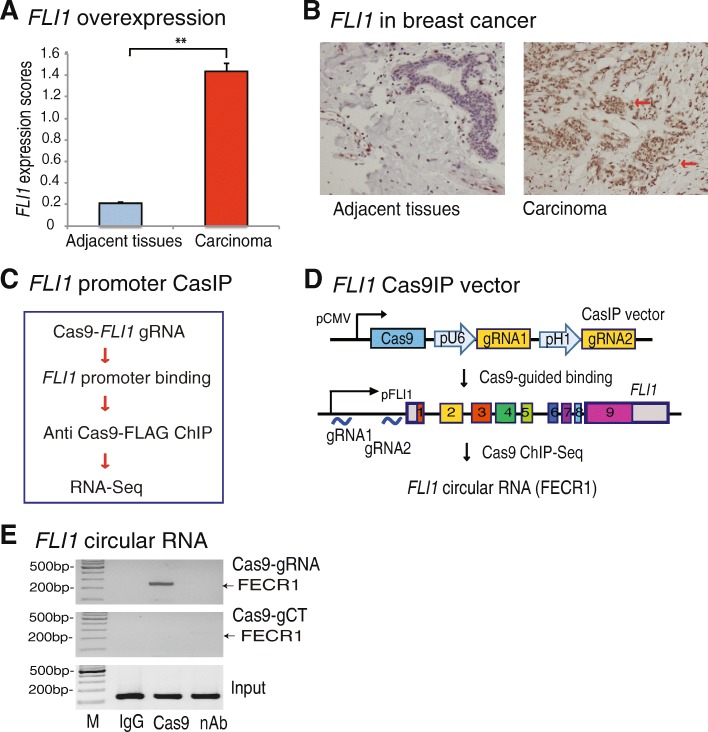
Identification of *FLI1* circular RNA by Cas9IP. **a** Overexpression of *FLI1* in breast cancer tissues. *FLI1* expression was quantitated by immunohistochemical staining and was evaluated as the expression score. **p < 0.01 in breast carcinoma tissues as compared with their adjacent tissues. **b** High expression of *FLI1* in carcinoma as compared with adjacent tissues. Red arrow: dark brown immunohistochemical staining of *FLI1* oncoprotein. **c** CRISPR Cas9-guided chromatin immunoprecipitation (CasIP). Cas9, CRISPR Cas9; *FLI1* gRNA, Cas9 guiding RNAs that target the *FLI1* promoter. Cas9 binds to the *FLI1* promoter through a mechanism of base pairing between the gRNA and target DNA. After fixation, the Cas9-*FLI1* promoter chromatin complex was immunoprecipitated by an anti-Cas9 antibody. The CasIP-captured RNAs were sequenced to identify the RNA components that regulate the gene activity in breast cancers. **d** Identification of *FLI1* circular RNA by CasIP. In the *FLI1* Cas9-gRNA cassette vector, two Cas9 gRNAs are transcribed by U6 and H1 promoters, respectively, and guide Cas9 to the *FLI1* promoter. The CasIP sequencing identifies a novel *FLI1* exonic circular RNA that interacts with the gene promoter. **e** Enrichment of FECR1 in the *FLI1* promoter. After CasIP, the captured RNAs were reverse transcribed to quantitate the abundance of FECR1 in the Cas9-captured promoter complex. M, 100 bp marker; IgG, ChIP with antibody control; Cas9, ChIP with anti-Cas9 antibody; nAb, the ChIP negative control, in which the anti dCas9-FLAG antibody was replaced by the equal amount of albumin protein

To determine the role of *FLI1* in breast cancer, we first examined its expression in tumor samples collected from patients with breast cancer. Using immunohistochemical staining, we found that *FLI1* was significantly activated in breast tumor tissues as compared with adjacent normal tissues (Fig. 1a, b; red arrows). There was also a relatively high abundance of *FLI1* in breast cancer tissues associated with metastases (Additional file 1: Figure S1A-S1B) and in advanced stages (Additional file 1: Figure S1C-S1D).

To identify the molecular components that aberrantly regulate *FLI1* in breast cancer, we utilized a novel CasIP approach to immunoprecipitate the *FLI1* promoter chromatin complex (Fig. 1c). We hypothesized that identification of the components that participate in the formation of the chromatin interaction network in the gene promoter might suggest mechanisms underlying the aberrant *FLI1* activation in tumors.

We constructed a lentiviral vector containing the catalytically inactive CRISPR Cas9 (dCas9) and two *FLI1* promoter gRNAs (Fig. 1d and Additional file 1: Figure S2) and transfected it into MDA-MB231 breast cancer cells. After puromycin selection, stable cells were treated with 1% formaldehyde to fix the dCas9-gRNA-*FLI1* promoter chromatin complex. To avoid RNA degradation, we performed an in situ reverse transcription to convert chromatin RNAs into cDNA with biotin-dCTP. The *FLI1* chromatin complex was immunoprecipitated with an anti Cas9-FLAG antibody. After reversing the crosslinks, the *FLI1* promoter-associated cDNAs were separated from genomic DNAs with streptavidin beads and were cloned into pJet vector for sequencing. Through this Cas9 immunoprecipitation sequencing, we identified a novel *FLI1* circular RNA that interacted with the *FLI1* promoter that we refer to as FECR1 (*FLI1* exonic circular RNA).

The interaction of FECR1 with the *FLI1* promoter was confirmed using CasIP PCR. We found that FECR1 was enriched in breast cancer cells that were transfected with Cas9-*FLI1* gRNAs (Fig. 1e, top panel, lane 2). No CasIP signals were detected in cells that were transfected with Cas9 control gRNAs (gCT, middle panel). Neither was the FECR1 signal detected in the IgG control (lane 1) and the non-antibody control (nAb, lane 3). Thus, FECR1 specifically interacted with the *FLI1* promoter in breast cancer cells.

### FLI1 circular RNA is derived from back splicing between exon 4 and exon 2

**Fig. 2:**
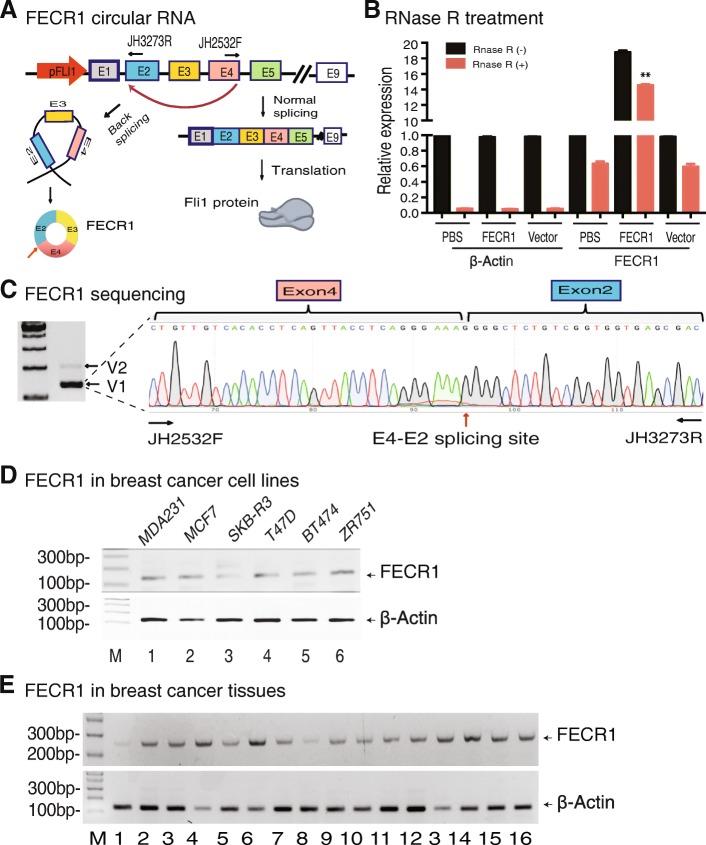
FECR1 is associated with the development of breast cancer. **a** Formation of *FLI1* exonic circular RNA. FECR1 is formed by back splicing of *FLI1* exon 4 to exon 2 and is composed of exons 4-2-3. **b** Detection of FECR1 circular RNA after RNase R digestion. RNA samples were treated with RNase R to remove linear RNAs. For qPCR normalization, the abundance of FECR1 was calculated by standardizing over the spike DNA control and setting the PBS control as 1. **p < 0.01 as compared with PBS and vector controls. **c** Sequencing of FECR1. Two FECR1 variants were detected by RT-PCR (left panel). V2 was expressed at very low level. We thus focused on the major form V1. The circular RNA (V1) was amplified with a forward primer (JH2532F) that is located at the end of exon 4 and a reverse primer (JH3273R) at the 5′-region of exon 2. Red arrow: back-splicing site. **d** Expression of FECR1 in breast cancer cell lines. **e** Expression of FECR1 in breast cancer tissues. Beta-actin was used as the PCR control

To delineate the structure of this newly identified circular RNA, we used a pair of circular RNA-specific PCR primers to amplify the cDNA fragment that covers the back-splicing site (Fig. 2a); with these primers, the linear *FLI1* mRNA would not be amplified.

To confirm the circularity of FECR1, we treated the total RNA from MDA-MB231 tumor cells with RNase R, which specifically digests linear RNA. After RNase R digestion, the expression of the circular RNA was detected by FECR1-specific PCR primers. After removal of linear RNAs, we still detected the presence of FECR1 in MDA-MB231 cells and FECR1-overexpressing MDA-MB231 cells (Fig. 2b). The *FLI1* linear mRNA was digested by RNase R, and only very weak singles were detected by PCR. These data further confirmed that FECR1 is a circular RNA.

To further characterize FECR1, we utilized PCR to amplify the major FECR1 isoform consisting of *FLI1* exons 4-2-3 (Additional file 1: V1, Figure S3A-S3B), and the minor FECR1 isoform contained *FLI1* exons 5-2-3-4 (Additional file 1: V2, Figure S3C-S3D). In support of this finding, the circular RNA sequencing database also showed the presence of FECR1 circular RNA (Additional file 1: Figure S4). Sequencing analysis revealed that FECR1 V1 was formed by back splicing between *FLI1* exon 4 and exon 2, and the V2 variant was jointed between exon 5 and exon 2 (Fig. 2c and Additional file 1: Figure S5). Since V2 was found in such low abundance, we focused on the isoform V1.

We also used RT-PCR to examine the expression of FECR1 in other breast cancer cell lines (Fig. 2d). FECR1 was also expressed in breast cancer tissue samples collected from patients at surgery (Fig. 2e).

### FECR1 promotes tumor cell invasion

**Fig. 3:**
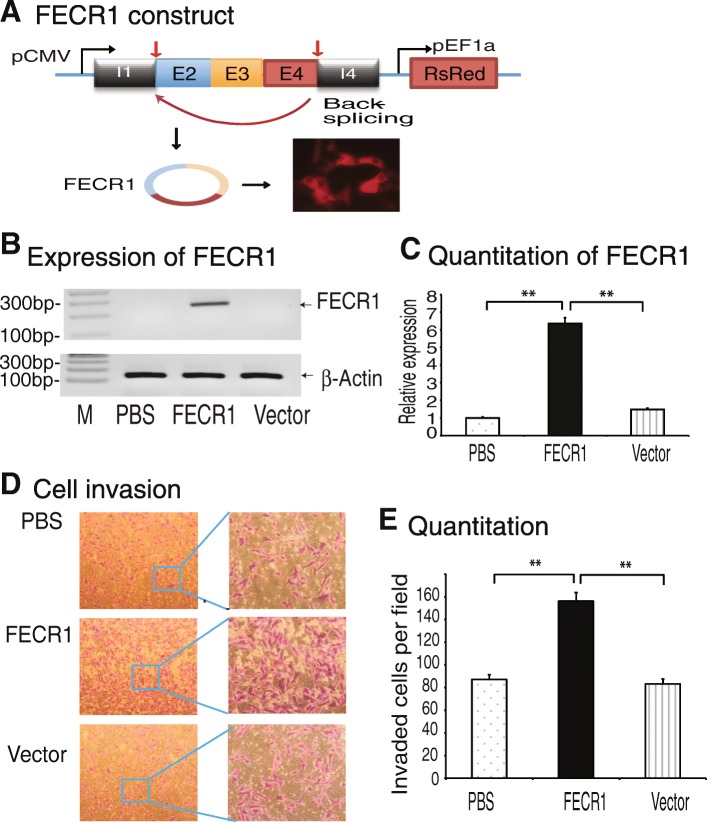
*FLI1* circular RNA promotes invasion of breast cancer cells. **a** Ectopic expression of FECR1 in MDA-MB231 breast cancer cells. The FECR1 expression cassette is composed of *FLI1* exons 4-2-3 and the intron fragments containing the back-splicing elements. pCMV, CMV promoter; pEF1a, EF1a promoter. DsRed fluorescent marker was used to track the transfection. **b** RT-PCR of FECR1. After stable transfection, cells were collected and FECR1 was amplified by PCR. PBS and vector, control groups. **c** Quantitation of FECR1 by qPCR. **p < 0.01 as compared with PBS and vector controls. **d** FECR1 promotes cell invasion. Cells that crossed through the collagen-coated membrane of the transwell were stained and photographed. **e** Quantitation of invading cells. All data shown are mean ± SEM from three independent experiments. **p < 0.01 as compared with PBS and vector control groups

To characterize its physiological function, we overexpressed FECR1 using a lentiviral FECR1 expression vector. The expression cassette was synthesized by including part of the intron 1 sequence carrying the splicing acceptor sequence, exon 2, exon 3, exon 4, and part of intron 4 containing the splicing donor sequence. The synthetic expression cassette was placed under the control of the CMV promoter. The expression of FECR1 was tracked by the DsRed fluorescence marker in the vector (Fig. 3a).

After puromycin selection, stable clones were selected. RT-PCR revealed the overexpression of FECR1 in transfected cells (Fig. 3b, lane 2). Quantitative PCR also validated the overexpression of this circular RNA as compared with vector controls (Fig. 3c).

Recently, we showed that knockdown of *FLI1* with small interfering RNAs significantly attenuated the potential for invasion of highly metastatic human breast cancer cells [16]. We thus examined if increased amounts of FECR1 enhances cell invasion. As seen in Fig. 3d, e, ectopic expression of FECR1 enhanced cell invasion as compared with the vector control. These data suggest that like *FLI1*, FECR1 circular RNA also has the ability to promote metastasis of breast cancer cells.

We also used shRNA to knockdown FECR1 in these tumor cells. As a control, cells were transfected with a random shRNA control. We found that knockdown of FECR1 with shRNA significantly inhibited the invasion of tumor cells (Additional file 1: Figure S6).

### RNA reverse transcription-associated trap identifies the FECR1 target genes

As FECR1 binds to the *FLI1* promoter, we examined the cellular distribution of FECR1 in MDA-MB231 tumor cells. Cytoplasmic RNA and nuclear RNA were separated and were reverse transcribed. Using quantitative PCR, we found that FECR1 was present in both the cytoplasm and nucleus (Additional file 1: Figure S7).

**Fig. 4:**
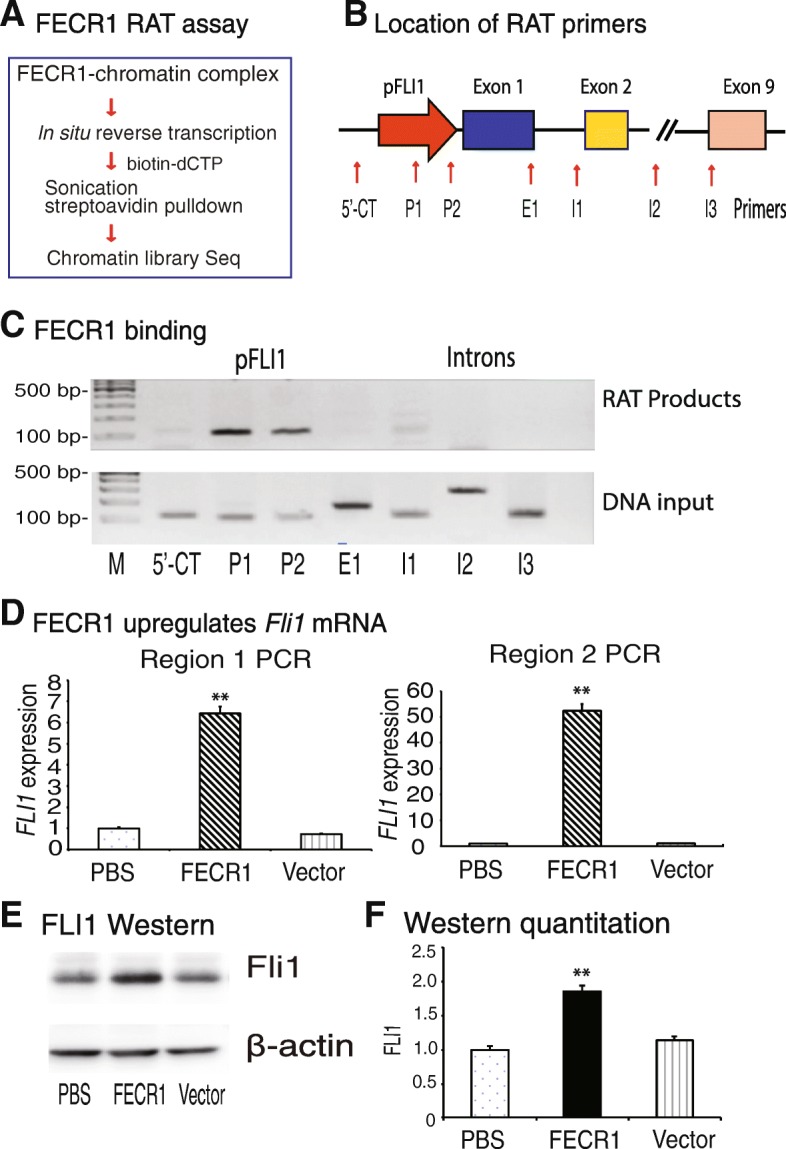
FECR1 upregulates *FLI1*. **a** Diagram of the RNA reverse transcription-associated trap (RAT) assay. FECR1 was in situ reverse transcribed using circular RNA-specific primers in the presence of biotin-dCTP. The FECR1-interacting chromatin DNAs were isolated for library sequencing. **b** Location of PCR primers to detect the interaction of FECR1 at the *FLI1* locus. 5′-CT, 5′-upstream control site. **c** Binding of FECR1 in the *FLI1* locus. The FECR1 RAT-captured chromatin DNAs were amplified by PCR using primers covering the *FLI1* locus. Note the binding of FECR1 in the *FLI1* promoter (P1, P2). **d** Activation of *FLI1* by FECR1. Expression of *FLI1* was quantitated by qPCR using two pairs of primers that cover different regions of *FLI1*. Region 1, the PCR product covers exon 4 to exon 6; region 2, the PCR product covers exon 3 to exon 4. **p < 0.01 as compared with PBS and vector control groups. Both qPCR data show that the overexpressed FECR1 upregulates the linear *FLI1* mRNA. **e** Western blot of *FLI1* protein. Cells that were stably transfected with FECR1-overexpression vector, vector control, and PBS were collected for Western blotting. **f** Quantitation of *FLI1* oncoprotein Western blot. **p < 0.01 as compared with PBS and vector control groups

We then used an RNA reverse transcription-associated trap (RAT) assay [18, 19] to identify a target gene network that interacts with FECR1 in addition to the *FLI1* promoter (Fig. 4a). After crosslinking, FECR1 was reverse transcribed in situ using a FECR1 circular RNA-specific complementary primer located at the exon 4-exon 2 splicing site (Additional file 2: Table S1 and Additional file 1: Figure S8). To reduce background from non-specific transcription, the reverse transcription reaction was performed at 65 °C using Maxima Reverse Transcriptase in the presence of biotin-dCTP. The FECR1 biotin-cDNA chromatin complex was pulled down with streptavidin beads. The FECR1-interacting target DNAs were eluted for library sequencing. The RAT-Seq bioinformatic analysis revealed that FECR1 circular RNA bound to multiple genes involved in pathways related to cell growth and proliferation (Additional file 1: Figure S9). Additional file 1: Figure S10 illustrates the partial FECR1 interactome of target genes.

We then designed a series of PCR primers to cover the *FLI1* gene locus (Fig. 4b) and examined the binding sites of FECR1. We found that FECR1 bound primarily to the promoter of *FLI1* (Fig. 4c, pFLI1, P1–P2). There was no binding of the circular RNA to remote intron regions (I1–I3) and the 5′-upstream control region (5′-CT).

FECR1 binds to the *FLI1* promoter in cis, and it is estimated that it may regulate its activity. To determine if FECR1 affects the activity of *FLI1*, we used two pairs of PCR primers (exons 4–6 and exons 3–4) to quantitate the expression of *FLI1* in the FECR1-expressing cell clones. As seen in Fig. 4d, quantitative PCR showed that ectopic expression of the circular RNA significantly upregulated *FLI1* as compared with the PBS and vector controls. Using Western blot, we found that overexpression of FECR1 significantly upregulated *FLI1* in the treated cells (Fig. 4e, f). These data suggest that FECR1 binds to the promoter of *FLI1*, where it activates the gene transcription.

### FECR1 induces extensive CpG DNA demethylation in the FLI1 promoter

**Fig. 5:**
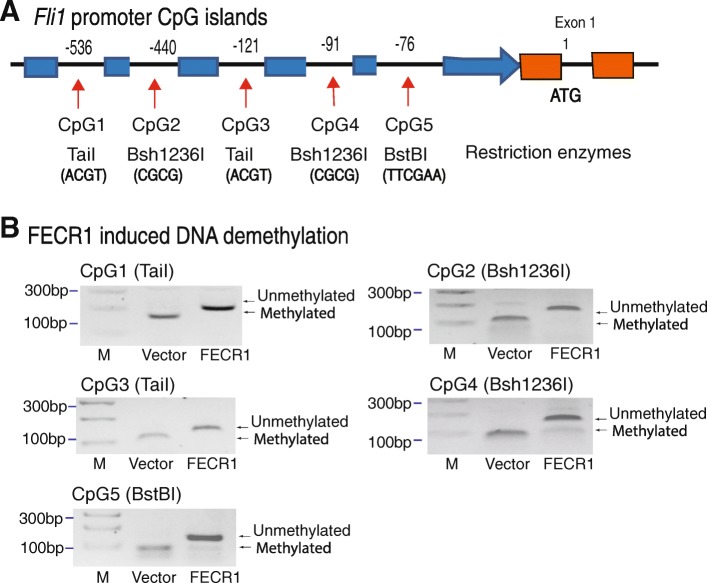
FECR1 induces DNA demethylation in the *FLI1* promote. **a** CpG islands in the *FLI1* promoter. In order to detect the status of DNA methylation in FECR1-expressing cells, we designed five pairs of methylation-specific primers located at each CpG island. Three restriction enzymes were used to separate methylated and unmethylated DNAs. **b** FECR1 induces DNA demethylation. DNA methylation was measured by combined bisulfite restriction analysis (COBRA). PCR products from FECR1-expressing cells and vector control cells were digested by TaiI, Bsh1236I, and BstB1 to separate the unmethylated and methylated DNAs. They recognize and digest the methylated ACGT, CGCG, and TTCGAA sites, respectively. After treatment with sodium bisulfate, unmethylated cytosines were converted to uracils, and the ATGT, TGTG, and TTTGAA sites are not digested by these enzymes. After digestion, unmethylated and methylated DNAs were separated on 3% agarose gels. Note the uncut demethylated bands in FECR1-expressing cells

We then examined the epigenetic mechanisms underlying the upregulation of *FLI1* by this circular RNA. The binding sequences of FECR1 to the *FLI1* promoter are very CpG-rich (Additional file 1: Figure S11). In order to learn if the *FLI1* promoter is epigenetically regulated by FECR1, we focused on the methylation status of the CpG islands in the proximal promoter of *FLI1* (Fig. 5a).

We used sodium bisulfite sequencing to assess DNA methylation in the *FLI1* promoter. After bisulfite treatment, three restriction enzymes were used to distinguish the methylated and unmethylated CpGs, including TaiI, Bsh1236I, and BstB1 (Fig. 5b). Bisulfite treatment converted the unmethylated CpGs into TpGs that will be not cut by these enzymes. Remarkably, we found that FECR1 induced extensive DNA demethylation when compared with the vector controls. Extensive DNA demethylation in the promoter correlates with the upregulation of *FLI1* in FECR1-expressing tumor cells.

### FECR1 promotes tumor metastasis by coordinately regulating DNMT1 and TET1

We then focused on the molecular mechanisms by which FECR1 epigenetically activates *FLI1*. DNMT1 is a key methyltransferase required for the maintenance of DNA methylation in mammals. It predominantly catalyzes methylation at hemimethylated CpG di-nucleotides [20]. Given the fact that FECR1 induces DNA demethylation in *FLI1* promoter, we examined whether DNMT1 was involved in this epigenetic control process.

**Fig. 6:**
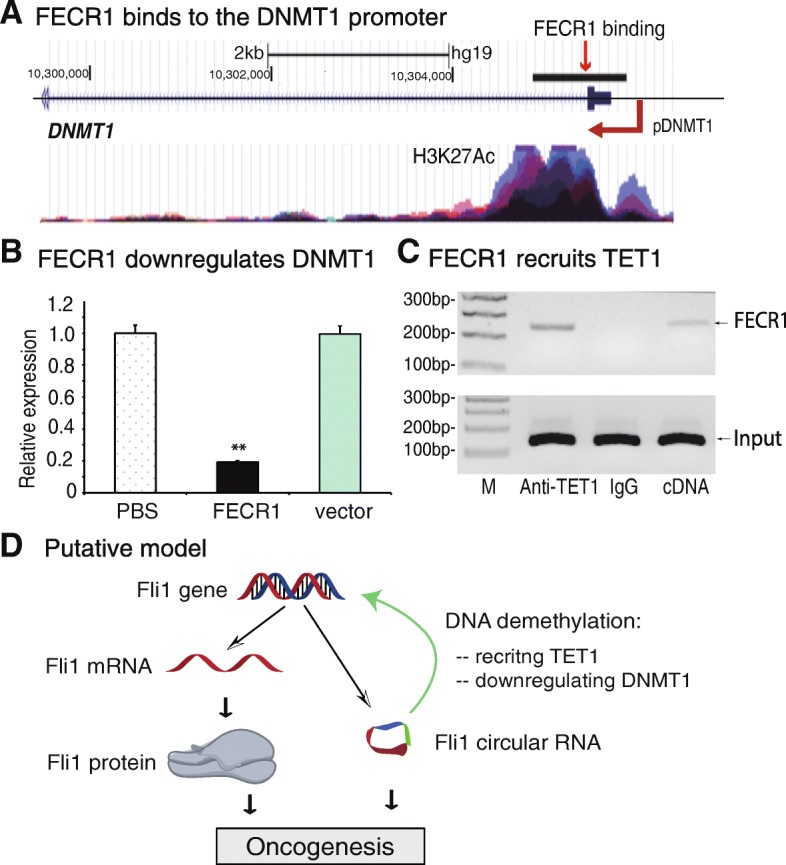
FECR1 coordinately regulates DNMT1 and TET1. **a** Binding of FECR1 to the DNMT1 promoter. After RAT sequencing, the FECR1 binding sequences were blasted to the human genome at the UCSC website. Histone 3 lysine 27 (H3K27) acetylation signal was also shown correspondingly. **b** FECR1 downregulates DNMT1. Expression of DNMT1 was measured by qPCR. **p < 0.01 as compared with PBS and vector control groups. **c** FECR1 recruits TET1 enzyme. RNA-chromatin immunoprecipitation (RIP) was performed to identify FECR1-TET1 binding. The TET-FECR1 chromatin complex was immunoprecipitated with an antibody against TET1. After removal of crosslinking, the immunoprecipitated RNAs were reverse transcribed, and the TET-interacting FECR1 was measured by PCR. IgG was use as the antibody control, and cDNA was used as the positive control. **d** Putative model of FECR1 in breast cancer. In addition to the conventional *FLI1* mRNA-oncoprotein model, *FLI1* also produces circular RNA FECR1. Through the interaction with *FLI1* promoter, FECR1 recruits TET1 demethylase and induces extensive DNA demethylation in the CpG islands. In addition, FECR1 also inhibits DNMT1, the critical enzyme that maintains DNA demethylation during DNA replication. Working together, FECR1 activates *FLI1*, which in turn promotes tumor cell invasion in breast cancers

First, we analyzed FECR1 RAT sequencing data and found that FECR1 was enriched in the promoter area of DNMT1, a methyltransferase that is essential for the maintenance of DNA methylation (Fig. 6a), where histone 3 lysine 27 (H3K27) is highly acetylated. We then quantitated the abundance of DNMT1 in treated cells. Ectopic expression of FECR1 significantly reduced the abundance of DNMT1 mRNA as compared with the controls (Fig. 6b). These data thus suggest that FECR1 binds to the DNMT1 promoter, where it downregulates DNMT1 transcription.

The status of DNA methylation in a given gene promoter is also determined by TET1, a Fe(II)/2-oxoglutarate-dependent dioxygenase. TET1 is a critical factor that induces the oxidation-deamination mechanism underlying active DNA demethylation [21, 22]. To determine if the binding of FECR1 to the *FLI1* promoter would regulate this demethylation process, we used an RNA-chromatin immunoprecipitation (RIP) method to pull down TET1-interacting complexes. The pulled-down RNAs were reverse transcribed and quantitated by PCR using primers specific for FECR1. Using this assay, we detected an enrichment of FECR1 in the TET1 antibody-precipitated complex (Fig. 6c).

To further confirm the interaction between FECR1 and TET1, we collected FECR1-overexpressing and vector control MDA-MB231 cells and performed chromatin immunoprecipitation with an antibody against TET1 (Additional file 1: Figure S12). Using PCR primers from the *FLI1* locus, we observed an enhanced binding of TET1 to the *FLI1* promoter (P1, P2) in FECR1-overexpressing cells as compared with that in the vector control group. Taken together, these data suggest that FECR1 circular RNA may regulate its target genes, like *FLI1*, by binding to the gene promoter and recruiting TET1 DNA demethylase to induce DNA demethylation.

We also used quantitative PCR to measure the expression of several other FECR1 target genes. We found that in addition to the upregulated *FLI1* and the downregulated *DNMT1*, FECR1 also significantly enhanced the expression of the *SERTED2* gene in FECR1-expressing cells (*P* < 0.01) (Additional file 1: Figure S13).

## Discussion

The present study demonstrates a non-canonical function for the *FLI1* in the development of breast cancer. We uncovered a novel pathway by which *FLI1* controls tumor metastasis in conjunction with its exonic circular RNA FECR1. To explore the molecular components that activate *FLI1* in breast cancer, we utilized a CasIP assay to identify a FECR1 circular RNA that interacts with the *FLI1* promoter. Increased expression of FECR1 enhanced tumor invasion in MDA-MB231 breast cancer cells. After binding to the *FLI1* promoter, the circular RNA recruited TET1 demethylase which demethylated the promoter CpG islands. FECR1 also bound to the DNMT1 promoter and downregulated its transcription although the specific mechanisms were unknown. Together, FECR1 induced extensive DNA demethylation in the *FLI1*, thereby enhancing its expression to promote metastasis (Fig. 6d). In a related study, we recently found that knockdown of FECR1 with shRNA significantly inhibited tumor metastasis and prolonged tumor survival in a small cell lung cancer xenograft model in nude mice [23].

*FLI1* is a member of the ETS family of transcription factors, sharing an ETS domain that binds specifically to their target promoters. Increased expression of *FLI1* is associated with poor survival in cancer patients [24]. It is now clear that *FLI1* promotes tumor growth by regulating a variety of target genes, including *Bcl-2* in the regulation of apoptosis, *gata-1* and *RB1* in erythroid differentiation, *MDM2* in the destabilization of the anti-apoptotic protein *P53*, VEGF-A in angiogenesis, and the *Notch1* signaling pathway [1, 11, 25–28]. Additionally, the oncoprotein also negatively regulates tumor suppressors, including *TP53* and *RB1*. Overall, like other members of the ETS family of transcription factors, *FLI1* regulates the expression of oncogenes, tumor suppressor genes, and other genes involved in blood vessel formation, invasion, and metastasis. It is conventionally believed that the proto-oncogene functions primarily through the expression of its oncoprotein product.

In this study, however, we have identified a new player in this malignant cascade. FECR1 circular RNA, a transcriptional byproduct of the proto-oncogene, promotes tumor metastasis at the level of gene transcription. FECR1 is a 571-bp circular RNA, consisting of *FLI1* exons 4-2-3. Using RNA reverse transcription-associated trap (RAT) sequencing, we demonstrate that this circular RNA binds to a variety of target genes that are involved in cell proliferation, transformation, apoptosis, and tumorigenesis. Notably, FECR1 binds to its own gene, *FLI1*, in cis and recruits TET1 demethylase. TET1 (ten-eleven translocation 1) is a member of a DNA hydroxylase family that possesses enzymatic activity toward converting 5mC into 5-hydroxymethylcytosine, 5-formylcytosine, and 5-carboxylcytosine through three consecutive oxidation reactions [29–32]. By inducing DNA hypomethylation in the gene promoter, FECR1 activates *FLI1*. Thus, FECR1 activates a positive feedback mechanism which promotes oncogene transcription. Unlike the conventional *FLI1* oncoprotein that regulates its downstream target genes, FECR1 functions by targeting the *FLI1* gene transcriptionally via a cis mechanism.

We also demonstrate that FECR1 binds to the *DNMT1* promoter in trans. After promoter binding, FECR1 silences *DNMT1* gene activity. In mammals, the genomic methylation process is executed by DNA methyltransferases DNMT1, DNMT2, DNMT3a, and DNMT3b [21, 33, 34]. Among these three methyltransferases, DNMT3A and DNMT3B are referred to as de novo methyltransferases that are responsible for establishing DNA methylation patterns and genomic imprints during embryogenesis. DNMT1, on the other hand, is essential for the maintenance of DNA methylation in the genome. During DNA replication, DNMT1 recognizes semi-methylated CpG as substrate and restores the specific methylation pattern on the daughter strand in a full copy of the parental DNA [20, 35, 36]. In this study, we show that FECR1 significantly downregulates *DNMT1*. We predict that the downregulation of *DNMT1* by FECR1 may cause activation of many target genes, including *FLI1*, that are associated with tumor growth. However, it is unclear how the binding of FECR1 induces downregulation of *DNMT1*. Future studies are needed to address the specific mechanisms by which FECR1 downregulates its downstream target genes, like *DNMT1*.

Circular RNAs belong to the category of long noncoding RNAs. However, recent studies have demonstrated that circular RNAs are also translated into functional peptides. With a combination of bioinformatic tools, Legnini et al. found that zinc finger protein circular RNA cir-ZNF609 is associated with heavy polysomes and is translated into a protein that controls myoblast proliferation [37]. Using ribosome footprinting, Pamudurti et al. demonstrated that a group of circRNAs was associated with translating ribosomes [38]. Sequence analysis shows that FECR1 contains the translation initiation codon ATG for *FLI1*. If actively translated, it is predicted that FECR1 may produce a short truncated *FLI1* (53 aa). Alternatively, it may use its internal ATGs as the initiation site and produces several short peptides. Future studies should validate the presence of these circular RNA-derived peptides and evaluate their functions in breast cancer.

It should be noted that the *EWS*/*FLI1* t(11;22) translocation, although occurring in more than 80% of the cases with Ewing sarcoma/primitive neuroectodermal tumor (EWS/PNET), has not been reported in solid tumors. In the latter case, *FLI1* is activated primarily by gene upregulation. In breast cancer, aberrant expression of *FLI1* correlates with advanced stage, poor differentiation, lymph node metastasis, and disease-free survival [16, 39]. Knockdown of *FLI1* attenuates tumor metastasis through multiple pathways, including the Rho GTPase pathway [16] and the epithelial-mesenchymal transition pathway [39]. Thus, *FLI1* may function as a tumor enhancer or promoter in solid tumors. Future studies are needed to address whether the *FLI1* gene may undergo subtle genetic alterations in solid tumors, including breast cancer.

## Conclusion

We have discovered a novel epigenetic pathway by which *FLI1* contributes to tumor metastasis. We identified a novel *FLI1* circular RNA FECR1 that interacts with the *FLI1* promoter. FECR1 binds to the *FLI1* promoter in cis and recruits TET1 demethylase. FECR1 also downregulates DNMT1 in trans. Together, this circular RNA may regulate metastasis of breast cancer cells by coordinating DNA methylation and demethylation in target genes that are involved in tumor growth. These data thus suggest that *FLI1* circular RNA may serve as a potential therapeutic target in the development of therapeutic interventions for metastatic breast cancer.

## Materials and methods

### Cell culture

Human breast cancer cell lines (MDA-MB231, MCF7, SKBR3, T47D, BT474, ZR751) and viral packaging 293 T cells were purchased from ATCC (Manassas, VA). Cells were routinely maintained in DMEM medium (Sigma, MO) containing 10% (*v*/*v*) fetal bovine serum (Sigma, MO), 100 U/ml of penicillin sodium, and 100 μg/ml of streptomycin sulfate (Invitrogen, CA), at 37 °C in 5% CO2.

### Breast cancer tissue samples

The study protocol was approved by the Research Ethics Board of the First Hospital of Jilin University [16, 40]. Informed consent was obtained from patients with breast cancer. Formalin-fixed and paraffin-embedded tissues of breast cancer were obtained from the biological sample library of the First Hospital of Jilin University. The pathological diagnosis was made in accordance with the histological classification of tumors developed by the World Health Organization. Tumor stage was defined according to the American Joint Committee on Cancer/International Union Against Cancer classification system. Tumors were histologically graded according to the Elston and Ellis method.

### Immunohistochemical staining of FLI1 oncoprotein

Expression of *FLI1* in breast cancer tissues was evaluated by immunohistochemical staining as previously described [16]. Briefly, tissue slides were de-paraffinized, rehydrated, and incubated in 3% hydrogen peroxide for 15 min to block endogenous peroxidase activity. The slides were incubated with primary anti-FLI1 polyclonal antibodies (Neomarker) at 4 °C overnight. After washing with PBS, the secondary antibodies (biotinylated goat anti-rabbit immunoglobulin) and streptavidin peroxidase complex reagent were added. The FLI1 signal was visualized using Polink-2 HRP DAB Detection kit.

The abundance of FLI1 was quantitated by immunoreactivity scoring as evaluated by two independent investigators. The intensity of FLI1 staining was scored as 0 (negative), 1 (weak), 2 (moderate), and 3 (intense). The immunoreactivity score was calculated as the percentage of positive cells per field multiplied by the intensity of staining.

### Construction of plasmids and viral transfection

A modified Cas9-guided chromatin immunoprecipitation assay [41, 42] was used to identify components that bind to the promoter of *FLI1*. The U6-gRNA1-T5-H1-gRNA2 cassette was synthesized by linking two Cas9-*FLI1* gRNAs with the U6 promoter and H1 promoter. *FLI1*-gRNAs included *FLI1*-gRNA1 5′-GGGGTTGAGGACACGTGCTG-3′ and *FLI1*-gRNA2 5′-GAGCCAATATTCCGTAGCAT-3′. The expression cassette was ligated at the Pme I and Not I sites in the lenti CRISPR-EGFP sgRNA 2 vector (Addgene Plasmid #51761), in which the wild-type Cas9 was replaced with the catalytically inactive dCas9 (Additional file 1: Figure S2).

Lentiviruses were packaged in 293 T cells using lipofectamine 3000 (Invitrogen, USA) with 2 μg of lentiviral expression and packaging plasmids. Viral supernatants were collected at 24 and 48 h post transfection. After addition of polybrene (8 μg ml^−1^), the supernatants were added in MDA-MB231 cells for transfection.

### Matrigel invasion assay

Invasion assays were carried out using 6-well BD Biocoat Matrigel Invasion Chambers (BD Biosciences, San Jose, CA) according to the manufacturer’s recommendations [43]. Briefly, 1.25 × 10^5^ cells in 0.5 ml DMEM medium were added to the inner chambers of Matrigel-coated wells with DMEM medium containing 10% serum in the bottom chamber. The cells were incubated for 24 h at 37 °C, and cells that did not invade through the pores were removed by a cotton swab. Cells on the lower surface of the membrane were stained with crystal violet and counted.

### RNA preparation and real-time PCR

Total RNA was extracted by TRIzol reagent (Sigma, MO) from cells and stored at − 80 °C. RT-PCR reaction was performed with an Eppendorf Thermol Cycler. The target amplification was performed by PCR of 1 cycle at 95 °C for 5 min; 33 cycles at 95 °C for 20 s, 60 °C for 15 s, and 72 °C for 20 s; and 1 cycle at 72 °C for 5 min. Quantitative real-time PCR was performed using 2× HotSybr Real-time PCR Kit (Mclab, CA). The threshold cycle (Ct) values of target genes were assessed by quantitative PCR in triplicate using a sequence detector (ABI Prism 7900HT; Applied Biosystems) and were normalized over the Ct of the β-ACTIN control.

### Cas9-gRNA-guided chromatin immunoprecipitation

A Cas9-guided chromatin immunoprecipitation assay [41, 42] was used to identify components that bind to a target gene DNA fragment. In this study, we constructed the dCas9-FLI1 promoter-gRNA vector by cloning two *FLI1* promoter gRNAs into the dCas9-2x gRNA vector that contains a mutated Cas9 (dCas9) and the tandem U6 and H1 promoters. Specifically, two oligonucleotides covering guiding RNA (gRNA) from the *Fli1* promoter were synthesized and inserted immediately downstream of the U6 and H1 promoters, respectively, followed by the Cas9-hairpin RNA-(T)5 sequence.

The dCas9-Fli1 promoter lentivirus was produced in 293 T cells as previously described. The viral supernatants were filtered through a 0.45-μm filter, concentrated by a PEG-IT kit (SBI, CA), aliquoted, and stored in a − 80 °C freezer. An aliquot of the Cas9-Fli1 promoter-gRNA lentivirus was used to transfect MDA 231 cells. The stable cells were selected in 1 μg/ml puromycin and collected for immunoprecipitation following the method as described previously. In brief, cells were fixed with 1% formaldehyde and sonicated for 180 s (10 s on and 10 s off) on ice with a Branson sonicator with a 2-mm microtip at 40% output control and 90% duty cycle settings. The sonicated chromatin TNAs containing dCas9-gRNA-Fli1 promoter complex were immunoprecipitated with Cas9 antibody (#ab191468, Abcam, MA). After reversal of cross-linking and proteinase K treatment, the Cas9-bound chromatin DNA and RNA were released and subjected to DNA/RNA sequencing and analyses.

### Identification of the FECR1 target genes by RNA reverse transcription-associated trap (RAT)

A RAT assay [18, 44] was modified to identify the interacting target genes of FECR1. Specifically, cells were cross-linked with 2% formaldehyde and lysed with hypotonic buffer (10 mM Hepes, pH 7.9, 1.5 mM MgCl2, 10 mM KCl, 0.4% NP-40, RNase inhibitor 100 U/ml, 1× protease inhibitors). Nuclei were suspended in 1× reverse transcription buffer. Reverse transcription was performed using FECR1-specific antisense primers and biotin-dCTP. To reduce non-specific reaction, the transcription was performed with Maxima Reverse Transcriptase (Thermo Fisher Scientific, CA) at 65 °C for 50 min. The reaction was stopped by adding 4 μl 0.5 M EDTA. After nuclear lysis, the chromatin complex was subjected to sonication for 180 s (10 s on and 10 s off) on ice with a Branson sonicator. The biotin-FECR1 cDNA/chromatin DNA complex was pulled down with biotin-streptavidin magic beads (Invitrogen, CA). After reversing the cross-links and washing with 10 mg/ml proteinase K at 65 °C for 2 h and treatment with 0.4 μg/ml RNase A for 30 min at 37 °C, the genomic DNA that interacts with the circular RNA was extracted and digested by Mbo I and ligated with the NEBNext adaptors (NEBNext® ChIP-Seq Library Prep Master Mix) to construct the library for Illumina sequencing (Shanghai Biotechnology, Shanghai). For the control group, a random RAT oligo (Additional file 2: Table S1) was used to generate RAT control library for Illumina sequencing.

### Confirmation of FECR1 as a circRNA by RNase R treatment

To confirm FECR1 as a circular RNA, the RNA samples were treated with RNase R to remove the linear RNA [37, 45]. The untreated RNAs were used as the control. For RNase R treatment, 1 μg of total RNA was treated with 2 U/μg of RNase R (Epicentre Technologies, Madison, WI), at 37 °C for 30 min. For qPCR normalization, 1 pg spike-in DNA was added to each reaction [37]. Following phenol/chloroform/ethanol extraction, RNAs were converted into cDNAs by reverse transcription and quantitated by qPCR using FECR-specific primers. The abundance of FECR1 was calculated by standardizing over the spike-in DNA control [37].

### RAT-seq data analysis

Raw data were filtered with Fastx (version: 0.0.13), and approximately 12 million single-end reads of 50 bp length from RAT-seq were mapped to hg38 reference genome using bowtie2 (version 2.2.5) with default parameters [46]. Only reads with at most one mismatch and a Phred quality score greater than or equal to 30 were kept for further analysis. Multiple reads that map to the exact same coordinates were further filtered out as PCR duplicates. The remaining high-quality mapping reads were extended to 500 bp fragments, which were then used to compute genome-wide coverage at 5 kb resolution with *bedtools genomecov* function [47]. The resulting coverage tracks (bedgraph file) were visualized in the UCSC genome browser.

### DNA methylation analysis

Total cellular DNAs were extracted by Genomic DNA Purification Kit (Thermo Scientific, CA) and treated with sodium bisulfite using EZ DNA Methylation™ Kit (Zymo, CA) [48]. Bisulfite-treated DNA was amplified by polymerase chain reaction (PCR) under liquid wax in a 6-μl reaction containing 2 μl of 3× Klen-Taq I Mix, 2 μl template DNA, and 1 μl of each 2.5 μM primer. After incubation at 95 °C for 5 min, DNA was amplified by 38 cycles of 95 °C for 20 s, 60 °C for 20 s of annealing and 72 °C for 20 s of extension, and finally with extension at 72 °C for 5 min. Methylation PCR primer sequences are listed in Additional file 2: Table S1.

The status of DNA methylation was determined by restriction enzyme digestion [49]. Genomic DNA was treated with sodium bisulfite to convert the unmethylated cytosines into uracils. The treated genomic DNA was purified and amplified by PCR. After bisulfite treatment and PCR reaction, unmethylated CpGs will be converted into TpGs, while methylated CpGs remain unchanged. Three restriction enzymes (TaiI/Bsh1236I/BstBI) were used to separate the methylated and unmethylated CpG DNAs. Briefly, PCR DNAs were digested by TaiI/Bsh1236I/BstBI at 37 °C for 30 min and separated on 2% agarose gel. TaiI recognizes the ACGT site, Bsh1236I recognizes the CGCG site, and BstBI digests the TTCGAA site. The unmethylated CpGs were not to be digested by these restriction enzymes.

### Chromatin immunoprecipitation (ChIP) assay

ChIP assay was used to examine the binding of TET1 to the *FLI1* promoter and was performed using the method as described previously [50]. In brief, FECR1-overexpressing and vector control cells were fixed with 1% formaldehyde, sonicated on ice for 180 s (10 s on and 10 s off), and immunoprecipitated with an anti-TET1 antibody (Invitrogen, CA). The isotype-matched was used as the ChIP control. An aliquot of cell lysates was served as the input DNA control. Precipitated DNA was subjected to quantitative PCR analysis. The qPCR data were first adjusted over the input and were further standardized over the IgG control [43].

### RNA-chromatin immunoprecipitations (RNA ChIP)

RNA-CHIP was performed based on the protocol by Zhang et al. with minor modifications [51]. MDA-MB231 cells were transfected with FECR1 vector and control vector; RNA immunoprecipitation was performed utilizing reversible chemical crosslinking of RNA-protein interactions by formaldehyde followed by immunoprecipitation using a TET1 antibody (Invitrogen, CA). After immunoprecipitation, extracts were reverse crosslinked; total RNA was extracted using TRIzol reagent (Sigma, MO) and treated with RNase-free DNase I (Invitrogen, CA). RT-PCR was conducted using either random-hexamer as the manufacturer’s instructions (Invitrogen, CA). PCR was performed using a specific set of primers.

### Western blot analysis

Cells were lysed in RIPA buffer in the presence of the protease inhibitor cocktail and 1 mM phenylmethylsulfonyl fluoride. Equal amounts of protein were resolved by SDS-PAGE and subjected to Western blot analysis using enhanced chemiluminescence (Pierce). Antibodies to *FLI1* and β-actin were obtained from Abcam (MA, USA).

### Statistical analysis

Comparisons between groups were analyzed by *t* test. Comparison was made of groups with high *FLI1* expression (score > median score) and low *FLI1* expression (score ≤ median score). We assessed score comparisons between groups by one-way ANOVA test. Overall survival (OS) was calculated by using the Kaplan-Meier method, and the differences were assessed by using the log-rank test. *p* value of less than 0.05 was considered significant. Statistical calculations were performed using SPSS 13.0.

## Additional files

Additional file 1: **Figure S1.** *FLI1* overexpression in metastatic breast cancer tissues. **Figure S2.** Location of Cas9 gRNAs in the *FLI1* promoter. **Figure S3.** Sequences of FECR1 variants. **Figure S4.** FECR1 from circular RNA database websites. **Figure S5.** Sequencing of Fli1 circle RNAs. **Figure S6.** FECR1 knockdown inhibited cell invasion in MDA-MB231 cells. **Figure S7.** Cellular distribution of FECR1 circRNA. **Figure S8.** Location of the FECR1-specific RAT primer. **Figure S9.** FECR1 binding by RAT-Seq. **Figure S10.** The chromatin RAT interactome of the FECR1 circRNA. **Figure S11.** FECR1 binding sequences in the CpG-rich *FLI1* promoter. **Figure S12.** FECR1 enhances the binding of TET1 to the *Fli1* promoter. **Figure S13.** Expression of target genes in FECR1-overexpressing cells. (PDF 2053 kb)

Additional file 2: **Table S1.** Oligonucleotide sequences of PCR primers. (PDF 119 kb)
