# Supplementary material for: A novel FLI1 exonic circular RNA promotes metastasis in breast cancer by coordinately regulating TET1 and DNMT1
Source: Genome Biol. 2018 Dec 11;19:218. doi: 10.1186/s13059-018-1594-y (PMC6290540; doi:10.1186/s13059-018-1594-y)
Supplement: Supplementary file 1 — Figure S1. FLI1 overexpression in metastatic breast cancer tissues. Figure S2. Location of Cas9 gRNAs in the FLI1 promoter. Figure S3. Sequences of FECR1 variants. Figure S4. FECR1 from circular RNA database websites. Figure S5. Sequencing of Fli1 circle RNAs. Figure S6. FECR1 knockdown inhibited cell invasion in MDA-MB231 cells. Figure S7. Cellular distribution of FECR1 circRNA. Figure S8. Location of the FECR1-specific RAT primer. Figure S9. FECR1 binding by RAT-Seq. Figure S10. The chromatin RAT interactome of the FECR1 circRNA. Figure S11. FECR1 binding sequences in the CpG-rich FLI1 promoter. Figure S12. FECR1 enhances the binding of TET1 to the Fli1 promoter. Figure S13. Expression of target genes in FECR1-overexpressing cells. (PDF 2053 kb) [file 13059_2018_1594_MOESM1_ESM.pdf]

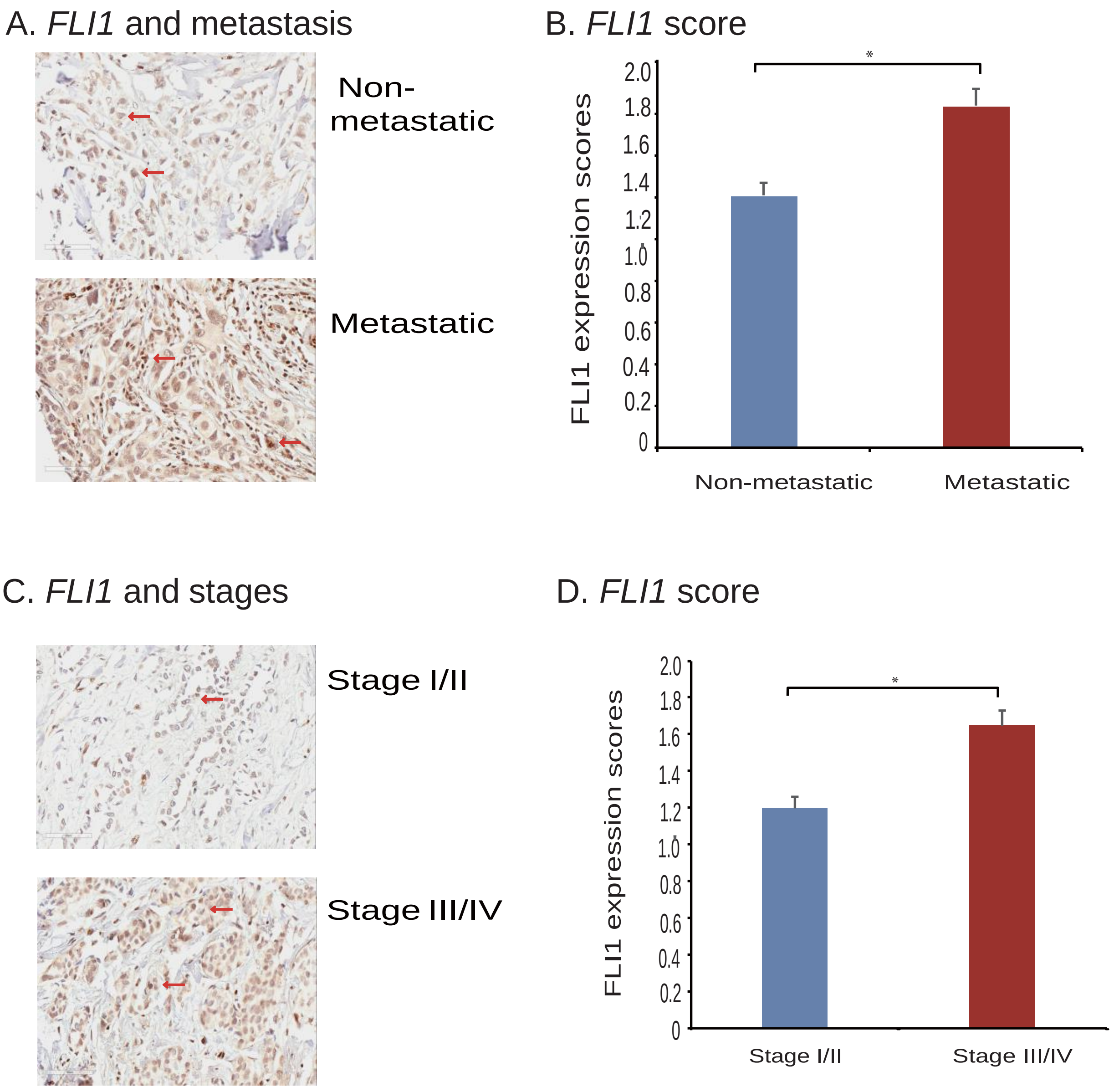

Figure S1. *FLI1* overexpression in metastatic breast cancer tissues.

- **A.** Overexpressed FLI1 in metastatic breast cancers. FLI1 oncoprotein was quantitated by immunohistochemical staining and was evaluated as the expression score. Red arrow: dark brown immunohistochemical staining of FLI1 oncoprotein.
- **B.** Quantitation of the FLI1 oncoprotein score between the metastatic and non-metastatic breast cancer tissues. * $p < 0.05$ between the two groups.
- **C.** High expression of *FLI1* in advanced stage of breast cancer. Red arrow: dark brown immunohistochemical staining of FLI1 oncoprotein.
- **D.** Quantitation of the FLI1 oncoprotein score in patients with advanced stage of breast cancer. * $p < 0.05$ between the two groups.

A. Cas9-IP vector

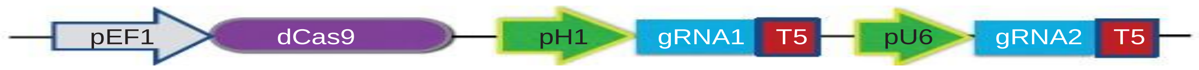

GATGAGTGGGTGAGCCGCTC   GTGGACCCCGTCATTGTTCC

B. Location of Cas9 *FLI1*-gRNAs

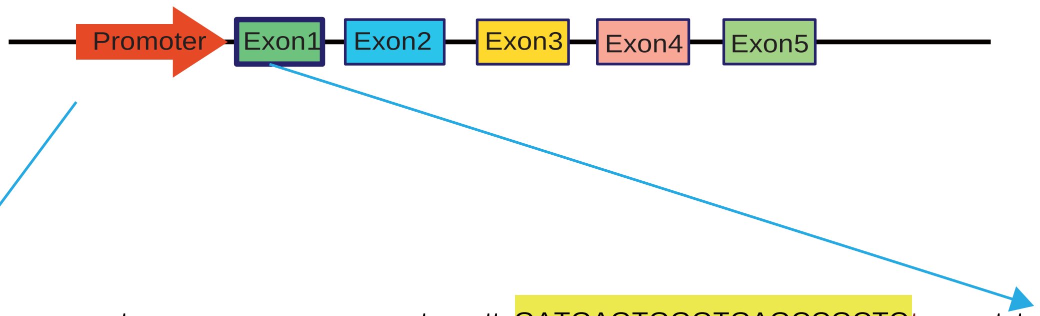

ccctgacagcgcgggtcagcccgaaaccgcccagaatccgcttcGATGAGTGGGTGAGCCGCTCtgggagtctg   gRNA1
ccgcgtctcggcagcggagggcctgagtggaaaaattcttattggatgcactttttaatggttggtttgcctcggttttcgtccgagtctt
ccccggcagtaggcgctgggttacccgcagccctagccaactctccctccatacccccctaccccacc cccaaaaagtttgtct
ccggctggaaaaagcattaatgaacgtatctgtgggaagaaacggaaagtgaatgagtcaggagggccaccttcggccaac
cgcctcaggaaaagcaaagaagccaaaggtctttggctttggattttgggggaaggtgatggggggaggttcagacttcggga
atcagggcgcggacgctgggcGTGGACCCCGTCATTGTTCCcggctagctcttatctcccaggagcaagtatcct   gRNA2
gtgtgcgcagcgcatgaatgtgtctgggcatctccgcgtatatttatatagtgtgtgatgcgaaaagcaggaccagcaggggagg
aagagggggtgtgggggggagggaagacgagggagagcagagggg**AGAGAAGAGAGAGGAGAGCTCG**   TS+1
AGGCGAGAGAGAGAGAGAGAGAGAGAGAGAGAGAGAGAGAGAGAGAGAGAGAGAGAGAGA   Exon1
TAGGACTTCCTC**CCCGATTCGCAAAGTGAAGTCACTTCCCAAAATTAGCTGAAAAAAAA**
GTTTCATCCGGTTAACTGTCTCTTTCGCTCCGCTACAACAACAAACGTGCACAGGGGAG
TGAGGGCAGGGCGCTCGCAGGGGGCACGCAGGGAGGGCCCAGGGCGCCAGGGAGG
CCGCGCCGGGCTAATCCGAAGGGGCTGCGAGGTCAGGCTGTAACCGGGTCAATGTGT
GGAATATTGGGGGGCTCGGCTGCAGACTTGGCCAAATGGACGGGACTATTAAG gtaagcg
gcggggcaacggacgcgggcggcggggaccggccggggaggc

Figure S2. Location of Cas9 gRNAs in the *FLI1* promoter.

A. The vector for CRISPR Cas9-guided chromatin immunoprecipitation (Cas9-IP). dCas9: catalystically inactive CRISPR Cas9; gRNA: Cas9 guiding RNAs that target the *FLI1* promoter (sequences under the diagram); pEF1: the human EF-1a promoter; pU6: U6 promoter; pH1: human H1 promoter; T5: the TTTTT termination signal of RNA polymerase III.

B. Location of the two *FLI1* promoter Cas9 gRNAs. The *FLI1* exon 1 mRNA was presented in green capital letters. Two Cas9 gRNAs were highlighted in yellow and PAM sequences in red in *FLI1* promoter region. TS+1: transcription initiation site.

A. FECR1 V1

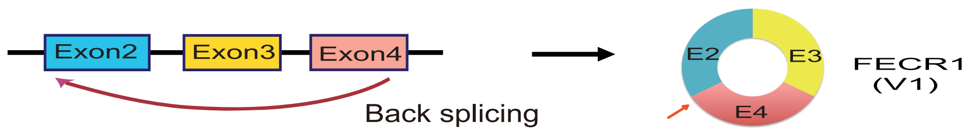

B. V1 sequence (exons 4-2-3)

ACCCCACACTGTGGACACAGGAGCATGTGAGGCAATGGCTGGAGTGGGCC Exon 4
ATAAAGGAGTACAGCTTGATGGAGATCGACACATCCTTTTTCCAGAACAT
GGATGGCAAGGAACTGTGTAAAATGAACAAGGAGGACTTCCTCCGCGCCA
CCACCCTCTACAACACGGAAGTGCTGTTGTCACACCTCAGTTACCTCAGG JH2532
GAAA**GGR**GGCTCTGTCGGTGGTGAGCGACGACCAGTCCCTCTTTGACTCA Exon 2
GCGTACGGAGCGGCAGCCCATCTCCCCAAGGCCGACATGACTGCCTCGGG
JH3273 GAGTCCTGACTACGGGCAGCCCCACAAGATCAACCCCCTCCCACCACAGC
AGGAGTGGATCAATCAGCCAGTGAGGGTCAACGTCAAGCGGGAGTATGAC
CACATGAATGGATCCA**G**GGAGTCTCCGGTGGACTGCAGCGTTAGCAAATG Exon 3
CAGCAAGCTGGTGGGCGGAGGCGAGTCCAACCCCATGAACTACAACAGCT
ATATGGACGAGAAGAATGGCCCCCCTCCTCCCAACATGACCACCAACGAG
AGGAGAGTCATCGTCCCCGCAG

C. FECR1 V2

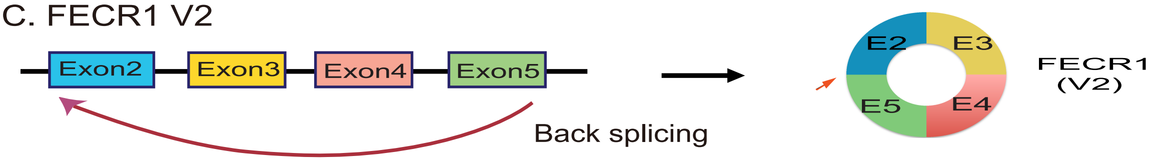

D. V2 sequence (exons 5-2-3-4)

JH3311 TTCACTGCTGGCCTATAATACAACCTCCCACACCGACCAATCCTCACGAT EXON 5
TGAGTGTCAAAGAA**GGR**GGCTCTGTCGGTGGTGAGCGACGACCAGTCCCT EXON 2
CTTTGACTCAGCGTACGGAGCGGCAGCCCATCTCCCCAAGGCCGACATGA
CTGCCTCGGGGAGTCCTGACTACGGGCAGCCCCACAAGATCAACCCCCTC
JH3273 CCACCACAGCAGGAGTGGATCAATCAGCCAGTGAGGGTCAACGTCAAGCG
GGAGTATGACCACATGAATGGATCCA**G**GGAGTCTCCGGTGGACTGCAGCG EXON 3
TTAGCAAATGCAGCAAGCTGGTGGGCGGAGGCGAGTCCAACCCCATGAAC
TACAACAGCTATATGGACGAGAAGAATGGCCCCCCTCCTCCCAACATGAC
CACCAACGAGAGGAGAGTCATCGTCCCCGCA**G**ACCCCACACTGTGGACAC EXON 4
AGGAGCATGTGAGGCAATGGCTGGAGTGGGCCATAAAGGAGTACAGCTTG
ATGGAGATCGACACATCCTTTTTCCAGAACATGGATGGCAAGGAACTGTG
TAAAATGAACAAGGAGGACTTCCTCCGCGCCACCACCCTCTACAACACGG
AAGTGCTGTTGTCACACCTCAGTTACCTCAGGGAAAG

Figure S3. Sequences of FECR1 variants.

A. Diagram of the formation of FECR1 V1 (major form). FECR1 V1 is formed by the back splicing between *FLI1* exon 4 and exon 2. The FECR1 beck slicing site is marked in red arrow.
B. Sequences of FECR1 V1 in the order of *FLI1* exon 4 (red) -exon 2 (blue) -exon 3 (yellow). The FECR1 V1 beck slicing site is marked with red arrow. The primer locations (JH2532, JH3273) are marked underline with the arrow indicting the orientation. R: A or G, a single nucleotide polymorphism at the beginning of exon 2.
C. Diagram of the formation of FECR1 V2 (minor form). FECR1 V2 is formed by the back splicing between *FLI1* exon 5 and exon 2. The FECR1 beck slicing site is marked in red arrow.
D. Sequences of FECR1 V2 in the order of *FLI1* exon 5 (green)-exon 2 (blue)-exon 3 (yellow)-exon 4 (red). The FECR1 V2 beck slicing site is marked bold with red arrow. R: A or G (single nucleotide polymorphism).

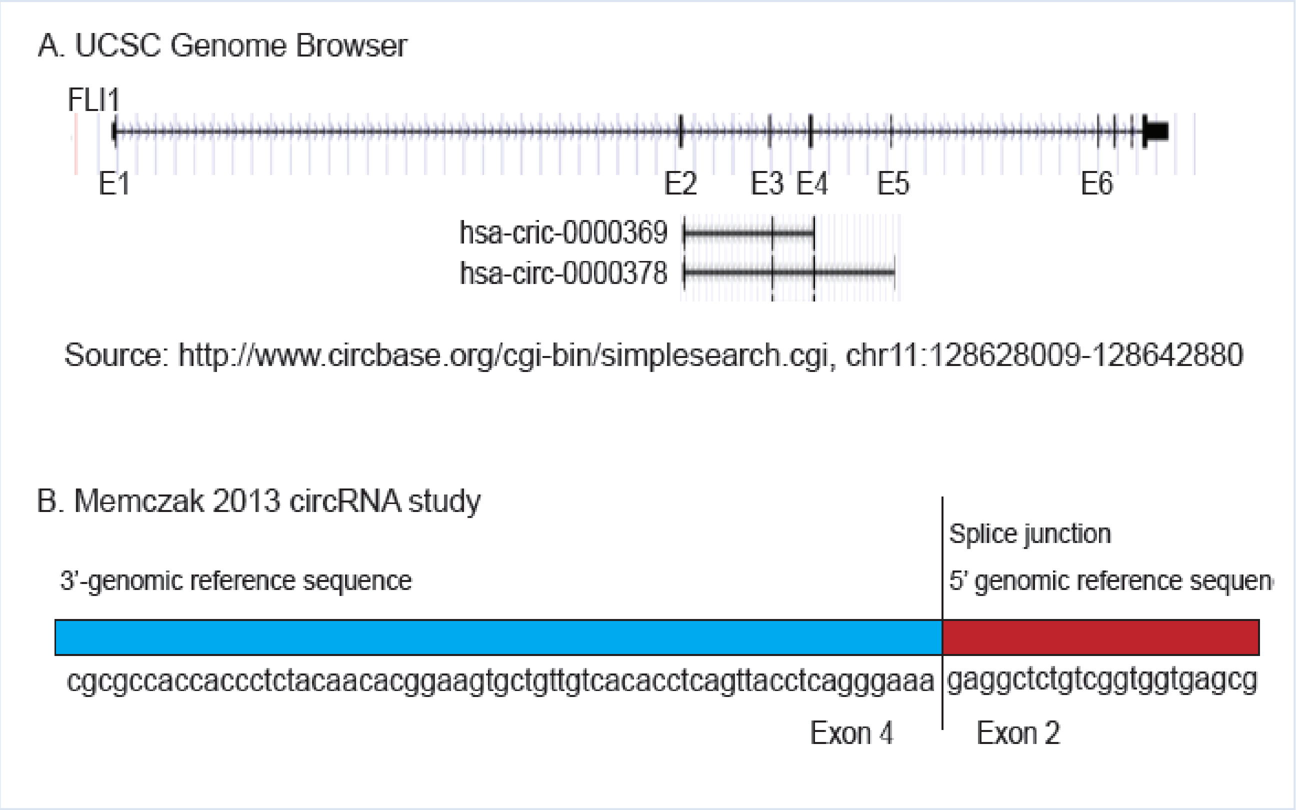

Source: http://www.circbase.org/cgi-bin/singlerecord.cgi?id=hsa_circ_0000369

Figure S4. FECR1 from circular RNA database websites.

A. Location of FECR1 V1 (hsa-cric-0000369) and V2 (has-cric-0000378) from UCSC genome browser. FECR1 V1 (hsa-cric-0000369) is composed of *FLI1* exons 4-2-3. FECR1 V2 (has-cric-0000378) is composed of *FLI1* exons 5-2-3-4.
B. The splicing junction sequence of FECR1 V1 (has-cric-0000369). The FECR1 V1 slicing junction site contains exon 4 (blue) and exon 2 (red).

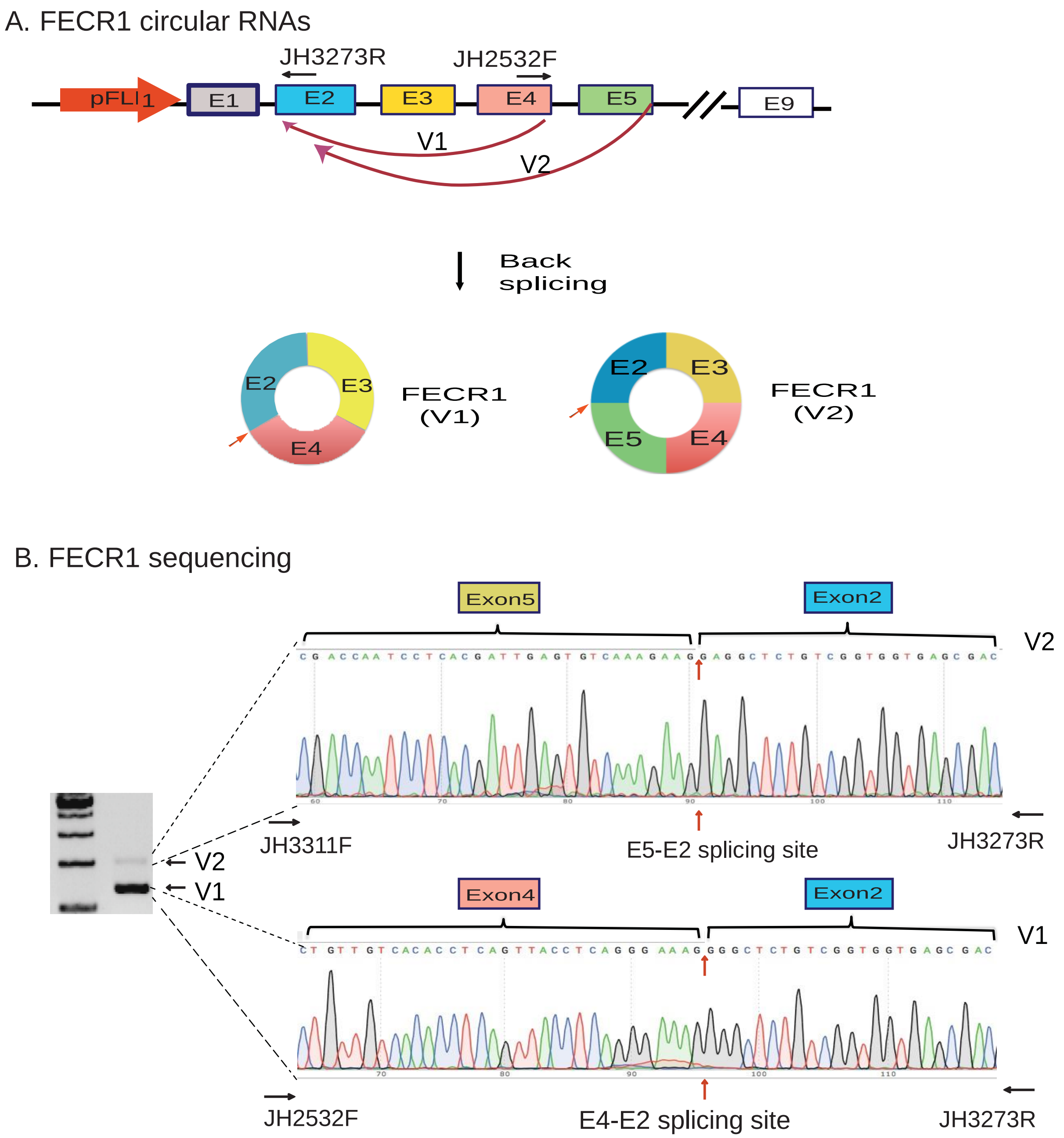

Figure S5. Sequencing of Fli1 circle RNAs.

A. Formation of FECR1 V1 and FECR1 V1 by the back splicing between *FLI1* exon 4 and exon 2, and exon 5 and exon 2, respectively. The FECR1 beck slicing site is marked in red arrow.

B. Sequencing of FECR1 variants. Red arrow indicates the site of back splicing between exon 5 and exon 2 for V2, and exon 4 and exon 2 for V1. Note the presence of a single nucleotide polymorphism (GGG vs GAG) at the beginning of exon 2 between V1 and V2.

A. Cell invasion in the rescue assay

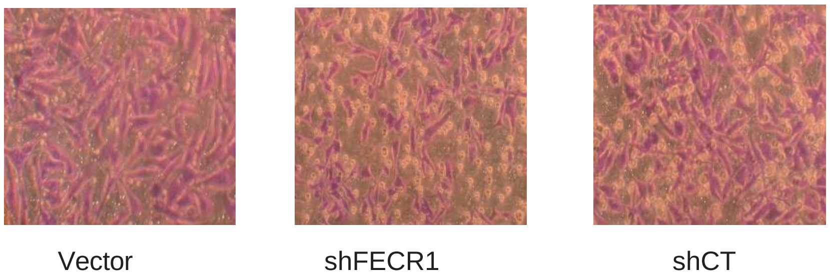

B. Quantitation

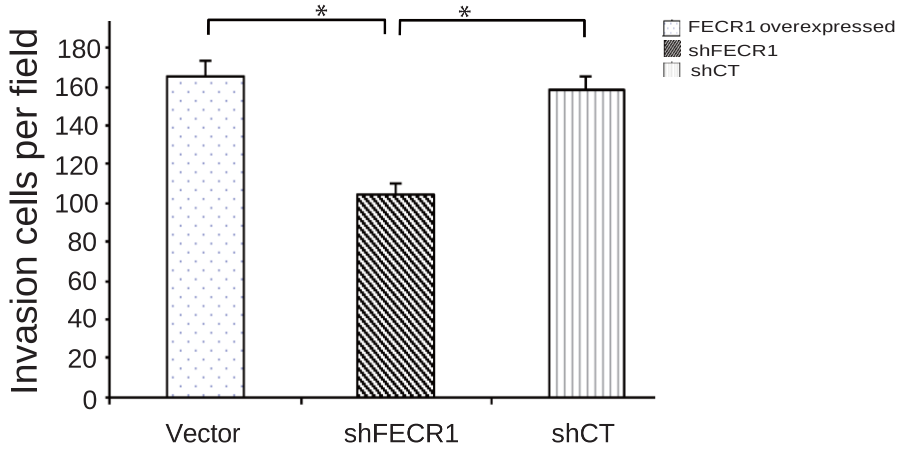

Figure S6. FECR1 knockdown inhibited cell invasion in MDA-MB231 cells.

A. Images of cell invasion. FECR1 was knocked down by FECR1-shRNA lentivirus (shFECR1) in the FECR1-overexpressing cells. Control cells were transfected by lentivirus carrying the shRNA control (shCT) or the lentiviral vector (Vector). Cells that crossed through the collagen-coated membrane of the transwell were stained and photographed.

B. Quantitation of invaded cells. Cells on the lower surface of the membrane were stained with the crystal violet and cells per filed were counted. All data shown are mean±SEM from three independent experiments. * $p<0.01$ as compared with the shCT control and vector groups.

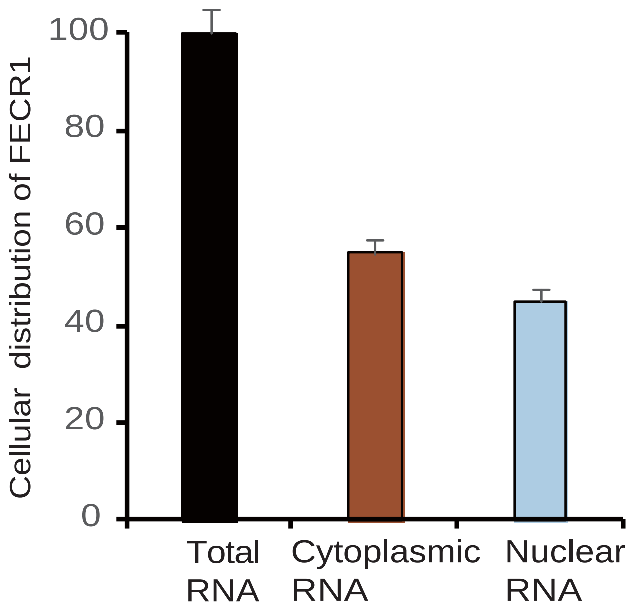

Figure S7. Cellular distribution of FECR1 circRNA.
RNAs were extracted from total cell, cytoplasm, and nuclei fractions. After reverse transcription, quantitative PCR was used to quantitate FECR1 abundance in each fraction. For comparison, the FECR1 circRNA in total cells was set as 100. All data shown are mean±SEM from three independent experiments.

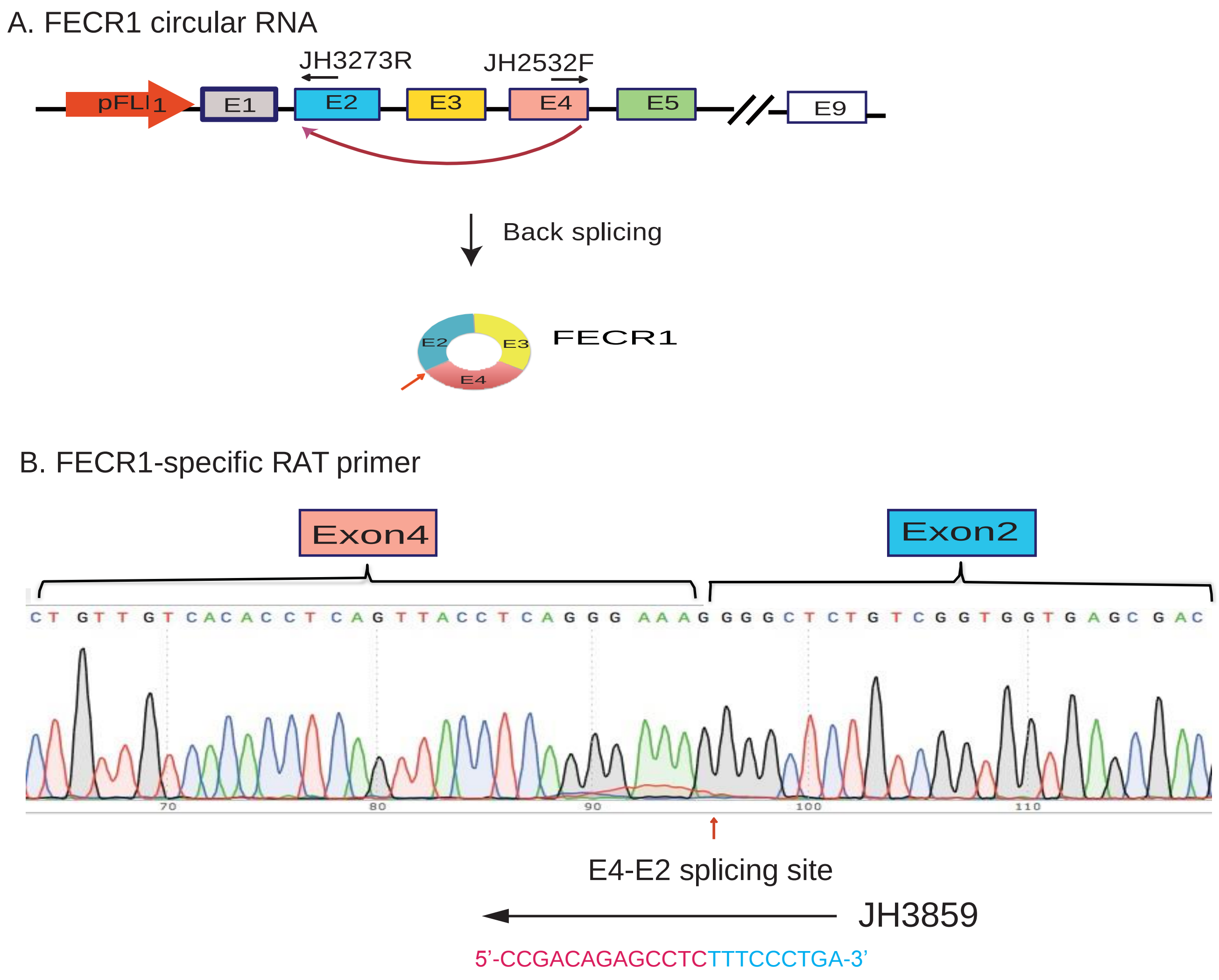

Figure S8. Location of the FECR1-specific RAT primer.

A. Diagram of FECR1 V1. FECR1 V1 is formed by the back splicing between *FLI1* exon 4 and exon 2. The FECR1 beck slicing site is marked in red arrow.

B. Location of primer JH3859. The primer is composed of DNA sequence from exon 4 (red) and exon 2 (blue). The FECR1 beck slicing site is marked in red arrow.

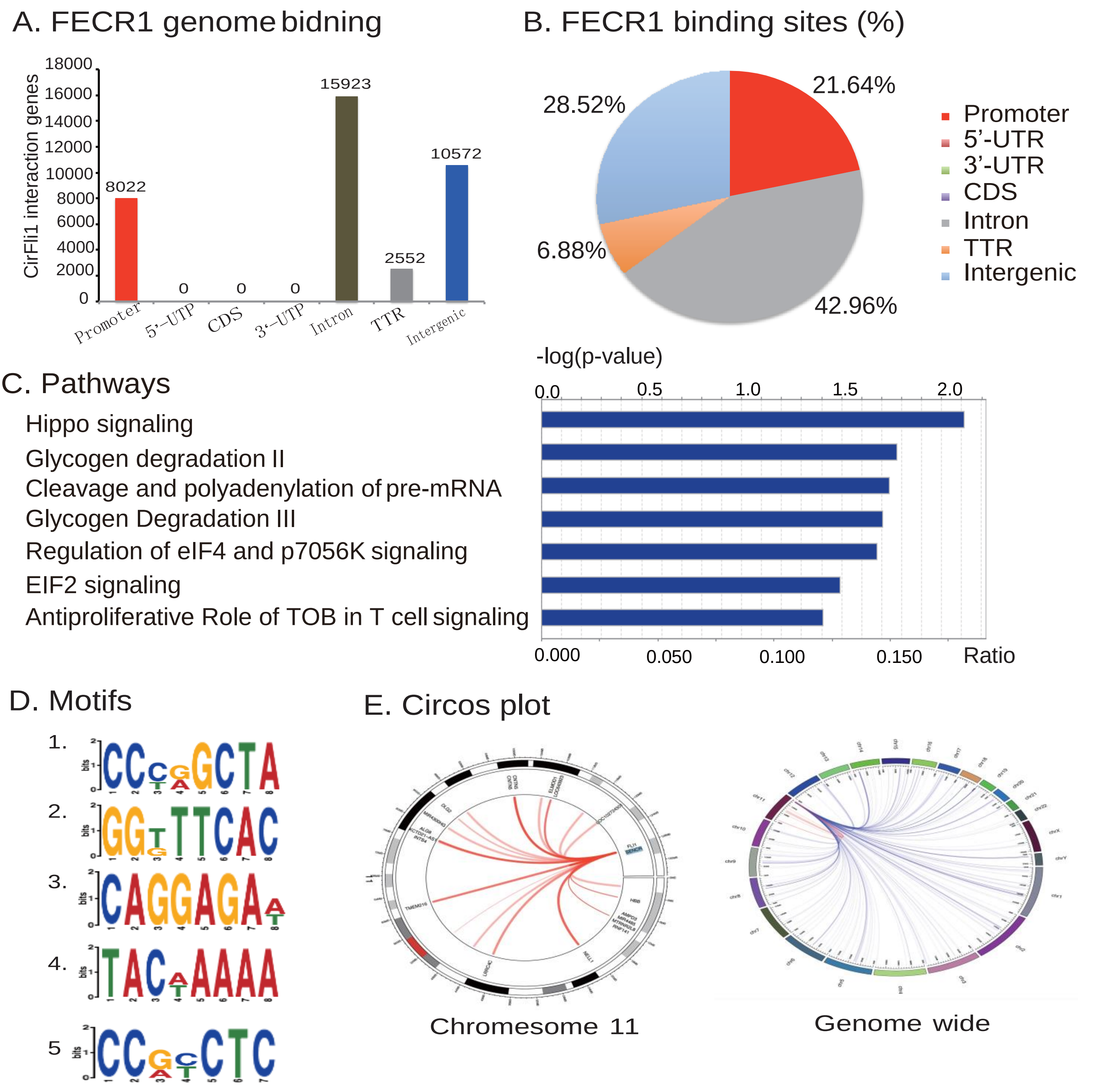

Figure S9. FECR1 binding by RAT-Seq.

A. Location of FECR1 binding in the genome. Most of FECR1 binding targets are located in gene promoters, introns, and intergenic regions.
B. Specific distribution (%) of FECR1 binding in the coding genes.
C. Gene ontology analysis of FECR1 target gene pathways. Bars: p-values of Fischer's exact test; squares (yellow): the ratio that represents the number of differentially expressed genes in a given pathway divided by total number of genes that make up that canonical pathway.
D. Consensus binding motifs of FECR1.
E. The Circos plot of FECR1 binding sites. In addition to the binding in chromosome 11, where the *FLI1* gene is located, FECR1 also binds to targets in other chromosomes.

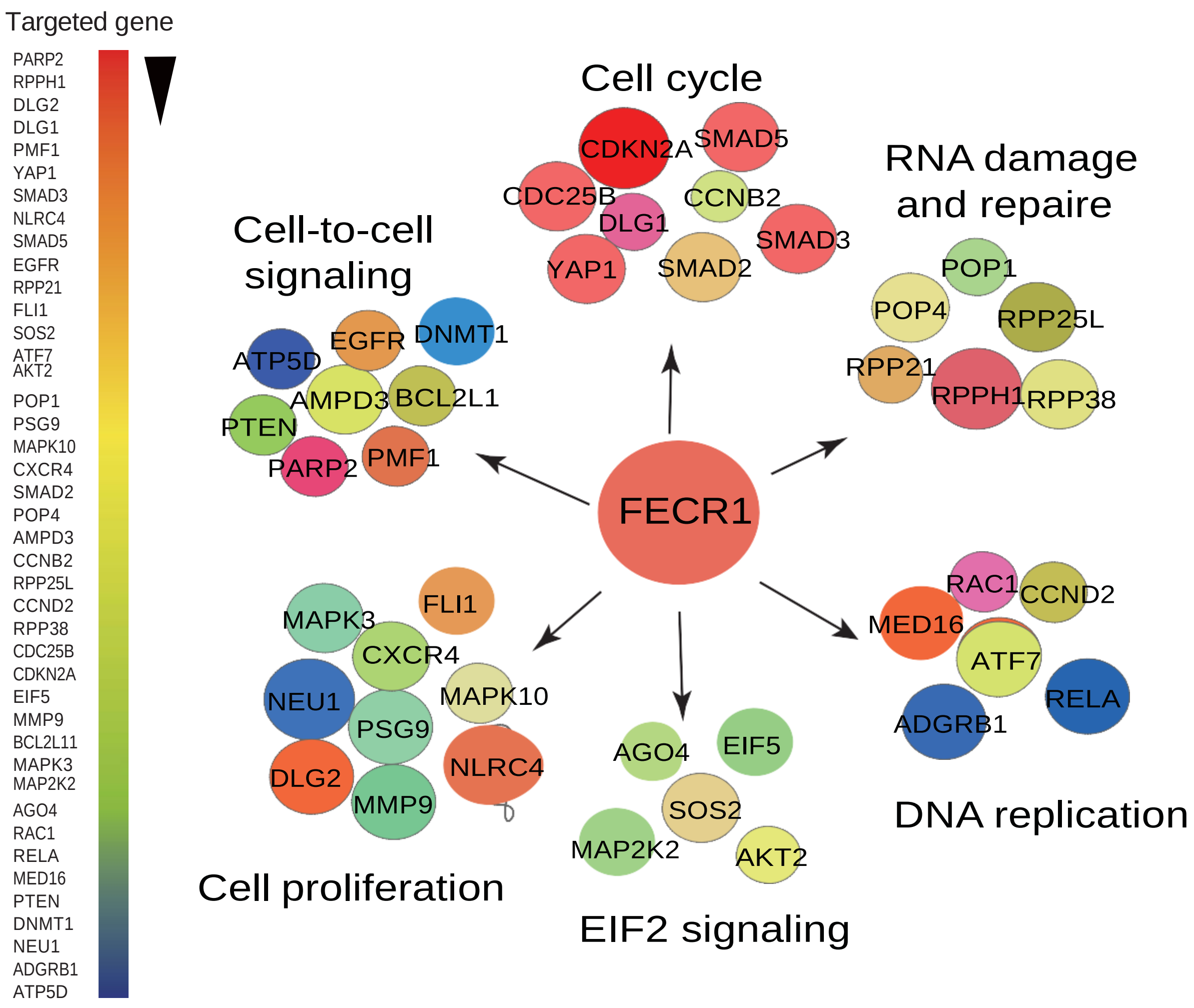

Figure S10. The chromatin RAT interactome of the FECR1 circRNA.

The top pathway binding genes were listed according to their enrichment value (FPKM).

A. *FLI1* promoter

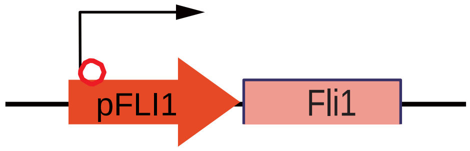

B. FECR1 binding sequences in the *FLI1* promoter (CpG islands)

```
AGAGAGAGAGAGAGAGAGAGAGAGAGAGAGAGAGAGAGAGAGAGAGATAGGA
CTTCCTCCCCGATTCGCAAAGTGAAGTCACTTCCCAAAATTAGCTGAAAA
AAAAGTTTCATCCGGTTAACTGTCTCTTTCGCTCCGCTACAACAACAAAC
GTGCACAGGGGAGTGAGGGCAGGGCGCTCGCAGGGGGCACGCAGGGAGGG
CCCAGGGCGCCAGGGAGGCCGCGCCGGGCTAATCCGAAGGGGCTGCGAGG
TCAGGCTGTAACCGGGTCAATGTGTGGAATATTGGGGGGCTCGGCTGCAG   Exon 1
ACTTGGCCAAATGGACGGGACTATTAAGgtaagcggcgggcaacggacg
cgggcggcggggaccggccgggaggcgaagcggcgggcggtaggtgcg
gggcccgcgtcccggaagacgtggcctctcccttccctccgcgcccccg
gcttcgcgccggctcctccggcgctcgggtggcgagtcctactcgcgggc
cagccggccaggcgcccgcattgagggcgaccctccccgaaatcccagc
ccccaaagtggagcccgcgcccccctcccacctcgctgcccgccgggct
gcaggaggggacggcgggaaaccggggaccccagggtacggaacggagt
caagggcgagagacctcgggtccaccactcccaccgacacccgggctc
ccgtggcccccagcgctaggcactgggcttcctctccgcagaggcggaaca
cggcggggggcggggctgcagtcgcccggctcgggtccctgcctggtgg
gccctggggtgggtaagtggcctcgttgcacgagggtaggggtgcaagtg
agtgtgtcggggatctatctgagcgggctcctccagatggaagcccctg
tttacatgtcagcgctttcccttcctctcggcactcagtcgcccggctct
aggcgctggagagccgccggctccgcgggactcctgggccggcctcgccg
cctctccggcggggaaccttccccagccccgtccgcacagatccctagc
gccccgagccccccgcccttcgcgcctaggcgtgcgccggctccaggacca
gggctcctggagctgtcgcctccgaaagggtcctgcgtccttcggagccg
cctccgttgcaggggccggctgtgagcccgcgcccccgcgcccgcccggcc
attcccaccccagccgccgccgccaagcctgggtctaggttgcaaagtgc
aacccaggcgg
```

Figure S11. FECR1 binding sequences in the CpG-rich *FLI1* promoter.

A. Location of FECR1 binding in the *FLI1* promoter.
B. FECR1 binding sequences in the *FLI1* promoter. CpG islands are underlined in red. FLI1 exon 1 was in blue capital letters and intron 1 was in small black letters.

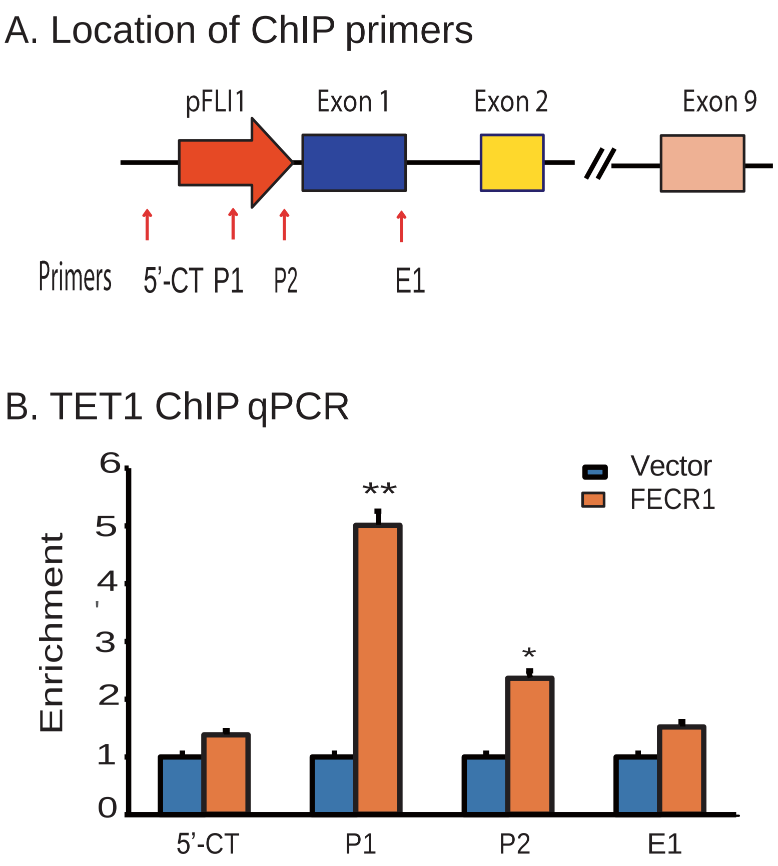

Figure S12. FECR1 enhances the binding of TET1 to the *Fli1* promoter.

A. Location of TET1 ChIP PCR primers. 5'-CT: 5'-upstream control site; P1-P2: *FLI1* promoter; I1-I3: intron control regions.

B. FECR1 enhances the binding of TET1 to the *FLI1* promoter. MDA-MB231 cells were transfected with FECR1 and vector lentiviruses. After puromycin selection, cells were collected for immunoprecipitation using an antibody against TET1. PCR was used to compare the enrichment of TET1 between the FECR1-overexpressing and vector control cells. The qPCR data were adjusted over the input and were further standardized over the IgG control. For comparison, the vector control value was set as 1 for each site in the *FLI1* locus. Note the enhanced binding of TET1 in the *FLI1* promoter (P1, P2) in FECR1-overexpressing cells.

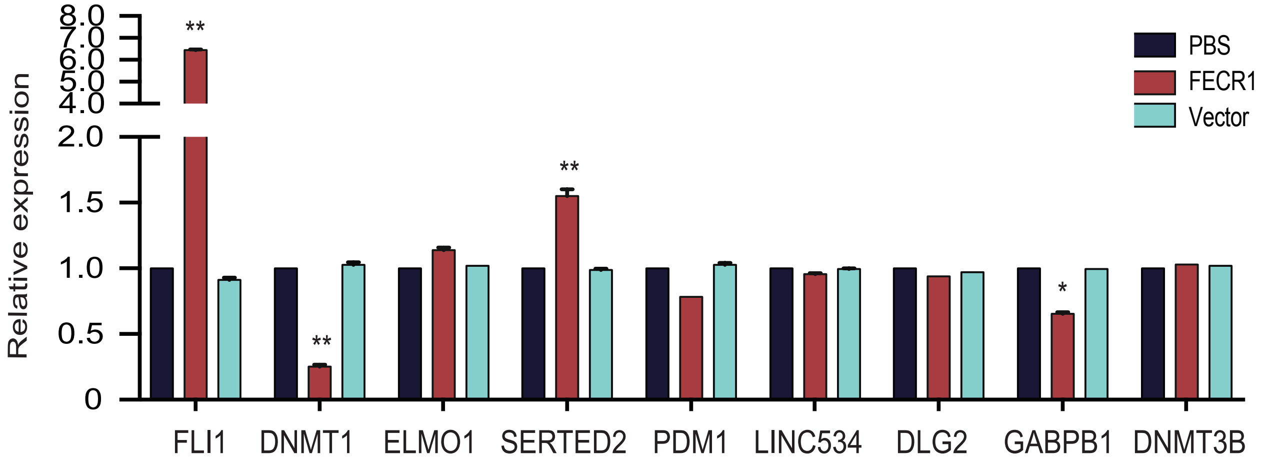

Figure S13. Expression of target genes in FECR1-overexpressing cells.
The mRNA abundance of FECR1 target genes were quantitated by real -time PCR. All data shown are mean±SEM from three independent experiments. * $p<0.01$ as compared with the PBS control and vector control groups.
