# Supplementary material for: A novel FLI1 exonic circular RNA promotes metastasis in breast cancer by coordinately regulating TET1 and DNMT1
Source: Genome Biol. 2018 Dec 11;19:218. doi: 10.1186/s13059-018-1594-y (PMC6290540; doi:10.1186/s13059-018-1594-y)
Supplement: Supplementary file 2 — Table S1. Oligonucleotide sequences of PCR primers. (PDF 119 kb) [file 13059_2018_1594_MOESM2_ESM.pdf]

Table S1. Oligonucleotide primers used for PCR

ID	Oligo Name	Oligo sequence	Product size
RT-PCR			
FLI (exon 2-3)	JH2530	TGACCACCAACGAGAGGAGAGTC	156bp
	JH2531	TACACAGTTCCTTGCCATCCATG	
FLI1 (exon 4-6)	JH2532	CTGTTGTCACACCTCAGTTAC	144bp
	JH2533	CATGTTATTGCCCCAAGCTCCTC	
FECR1	JH3273	AGTCAGGACTCCCCGAGGCAGT	137bp
	JH2532	CTGTTGTCACACCTCAGTTAC	
FECR1	JH3859	CCGACAGAGCCTCTTTCCCTGA	253bp
	JH2530	TGACCACCAACGAGAGGAGAGTC	
DNMT1	JH3540	GTCACCAACCCCGAGCCCATGG	193bp
	JH3541	CCAARGCCAATGGCTTTGGCCAG	
β-Actin	J880	CAGGTCATCACCATTGGCAATGAGC	135bp
	J881	CGGATGTCCACGTCACACTTCATGA	
GABPB1	JH4273	CCAGCTAAGAGACAATGTATCGA	159bp
	JH4274	GTAGGCCTCTGCTTCCTGTTCT	
SERTAD2	JH4277	CGTTAGTTCCCAGGTGGAGCT	128bp
	JH4278	AGACACCTTGGATGGACCGTCA	
DPM1	JH4279	GGGATGTTGCTGAACAGTTGGA	157bp
	JH4280	TGGTGTGAGAGATCAGCATCCAT	
ELMO1	JH4283	TTGCACTCCAGCATGCCGATAG	146bp
	JH4284	CATACTCGAGGACTGGATTCGT	
DLG2	JH4500	AGTCGGGTTGCTTGGGAATGCA	139bp
	JH4501	CACGCCCTCTTGTACTCTCTCT	
LINC00534	JH4496	GAGCCATTGCCTCTTGCTGTGA	151bp
	JH4497	CTGGAAAGGGAGTGGAACTGTGA	
DNMT3B	JH069	GCCCCCAA TCCTGGAGGCTAT	116bp
	JH070	CGCCTGTCAAGTCCTGTGTGTA	
circPVT1	NF063	CGACTCTTCCTGGTGAAGCATCTGAT	134bp
	NF064	TACTTGAACGAAGCTCCATGCAGC	
Spike-in DNA	JH1695	CGAGTACAAGCCCACGGTGC	124bp
	JH1696	GATGTGGCGGTCCGGATCGACG	
DNA methylation			
CpG1-2	JH5040	AGGTGATGGGGGGAGGTTTAGAT	242bp
	JH5041	ACCCCCTCTTCCTCCCCTACTAA	
CpG3-5	JH5044	GTGTATAGGGGAGTGAGGGT	285bp
	JH5045	ACCCCCCAATATTCCACACATTA	
FLI1 promoter binding			
1	JH4053	CTCGGTTTTCGTCCGAGTCTTC	119bp
	JH4054	TTTTTCCAGCCGGAGACAAACT	
2	JH2756	GGGCTGCGAGGTCAGGCT	106bp
	JH4528	GTTGCCCCGCCGCTTACCTTA	

3	JH4531	CTCTCCGCAGAGGCGGAACA	104bp
	JH4532	CTCGTGCAACGAGGCCACTTA	
4	JH2528	AGGGAGCTATAAGAGCCTAT	163bp
	JH2529	GAGACACTTGCATGAACACATC	
5	JH2657	GAACCTGACACTTCAGGCCA	120bp
	JH2758	AGGTAACTTAGGTGAATGAC	
6	JH2744	CTTTTGGGAATGGGCATAC	269bp
	JH2745	TCCATTCCCTAACCTGCGT	
7	JH3222	GCAAGTGGTTCTCATGGAAGC	126bp
	JH3223	ATGAGCCACCCAGTCTATGGTA	
Cas9-IP gRNA			
1		GATGAGTGGGTGAGCCGCTC	
2		GTGGACCCCGTCATTGTTCCCG	
control		GTTCCCTGCAAGAGTGCCCA	
RAT primers			
**FECR1**		AGTCCCTTTCTCCGAGACAGCC	
**RAT control**			
JH5849		ATGGACTGATGATCTTATGC	
JH5850		TACATAGTAGATCAGATACT	
